# Efficient Privacy-Preserving Access Control Scheme in Electronic Health Records System

**DOI:** 10.3390/s18103520

**Published:** 2018-10-18

**Authors:** Yang Ming, Tingting Zhang

**Affiliations:** School of Information Engineering, Chang’an University, Xi’an 710064, China; zhangting141012@163.com

**Keywords:** electronic health records, privacy preserving, access control, attribute-based signcryption, cuckoo filter

## Abstract

The sharing of electronic health records (EHR) in cloud servers is an increasingly important development that can improve the efficiency of medical systems. However, there are several concerns focusing on the issues of security and privacy in EHR system. The EHR data contains the EHR owner’s sensitive personal information, if these data are obtained by a malicious user, it will not only cause the leakage of patient’s privacy, but also affect the doctor’s diagnosis. It is a very challenging problem for the EHR owner fully controls over own EHR data as well as preserves the privacy of himself. In this paper, we propose a new privacy-preserving access control (PPAC) scheme for EHR. To achieve fine-grained access control of the EHR data, we utilize the attribute-based signcryption (ABSC) mechanism to signcrypt data based on the access policy for the linear secret sharing schemes. Employing the cuckoo filter to hide the access policy, it could protect the EHR owner’s privacy information. In addition, the security analysis shows that the proposed scheme is provably secure under the decisional bilinear Diffie-Hellman exponent assumption and the computational Diffie-Hellman exponent assumption in the standard model. Furthermore, the performance analysis indicates that the proposed scheme achieves low costs of communication and computation compared with the related schemes, meanwhile preserves the EHR owner’s privacy. Therefore, the proposed scheme is better suited to EHR system.

## 1. Introduction

With the speedy growth of new-generation information techniques like the cloud computing and Internet of Things, and the uninterrupted improvement of living standards of people, the concept of smart city has also got more attention. In particular, the electronic health records (EHR) system has been widely applied in smart city since its appearance, and it has gradually been developed and improved [1,2]. However, in face of the tremendous EHR data, a third-party platform is needed to store and manage these data. Cloud computing provides inexpensive distributed computing capabilities through the Internet, which has the characteristics of ultra-large-scale and low-cost. Hence, managing and storing the EHR data in cloud servers has become an inevitable trend. In EHR system, EHR owners generally upload and view their personal information, medical records and medication records from cloud servers. Storing the EHR data in cloud servers which improves the quality of personal medical health management while saving resources and reducing hospital expenses. Only authorized EHR users (such as doctors or nurses) are able to log in the cloud servers and access data.

Although there are many significant advantages when using cloud servers to manage the EHR data, it also brings some concerns, such as the security and privacy of the sensitive data [3,4,5]. If a malicious and unauthorized adversary breaks the EHR system and conducts a series of malicious actions, including leaking patient’s identity information and maliciously tampering with medical records, it will not only result in disclosure of patient personal privacy, but also lead to misdiagnosis by the doctors and brings serious consequences. Hence, it is necessary to put forward the access control requirements to legitimate users who can access the EHR data. Attribute-based encryption (ABE) is employed to supply fined-grained access control of the EHR data. The EHR owner defines the access policy to determine who is capable to obtain the EHR data and uploads them to the cloud servers after encrypting it using the access policy. The ciphertext could be decrypted simply if the attributes of the EHR user meet the access policy that is defined by the EHR owner. Such as, the encryption access policy is “Alice” ∨ “XXX Hospital ∧ Oncologist”. So, the EHR owner named “Alice” or the EHR user who is the “oncologist” in “XXX hospital” has the right to access the EHR data.

Although ABE schemes [6,7,8,9] could provide secure access control for the EHR data in EHR system, they still suffer from a serious problem that the access policy may leak EHR owner’s privacy. Here, the access policy will be send together with the ciphertext to EHR users in decryption phase, which may lead to the adversary gains owner’s related sensitive information from the access policy. This is caused by the construction of access policy is related to the EHR owner’s attributes. For instance, “Oncologist” is the sensitive information in the access policy for EHR owners. If anyone obtains this information, he might suspect that the EHR owner is suffering from oncology, which leads to the privacy leakage of the EHR owner. To achieve privacy-preserving for EHR system, some ABE schemes [10,11,12,13,14,15,16,17] were proposed.

However, all ABE schemes only support data encryption functionality and do not provide authentication capability. Attribute-based signcryption (ABSC) [18] mechanism emerges in integrating the fine-grained access control of data in attribute-based cryptography terminology and the efficient advantage of signcryption technology, which provides confidentiality, unforgeability and public verifiability simultaneously. Therefore, it is more appropriate to design a PPAC scheme for EHR system using the ABSC technology.

### 1.1. Our Contributions

In this paper, inspired by the ABSC mechanism and the cuckoo filter [19], a novel privacy-preserving access control (PPAC) scheme for EHR system is put forward. The major contributions are summed up as below:

- Based on the bilinear pairings, the ciphertext-policy attribute-based signcryption (CP-ABSC) scheme for EHR system is proposed. The proposed scheme ensures fined-grained access control of the EHR data, utilizes cuckoo filter to hide the access policy and preserves the privacy of EHR owners.
- The security analysis indicates that the proposed CP-ABSC scheme achieves the ciphertext indistinguishability and existential unforgeability in the standard model under the decisional bilinear Diffie-Hellman exponent (*q*-DBDHE) assumption and the computational Diffie-Hellman exponent (*q*-CDHE) assumption, respectively.
- The performance evaluation demonstrates that the proposed CP-ABSC scheme is more efficient than the related existing schemes [20,21,22,23] in terms of communication overheads and computation costs, and is right suitable for EHR system.

### 1.2. Organization

This paper is organized as below. The related work is described in Section 2. The preliminaries are reviewed in Section 3. The system model and security model are described in Section 4. The proposed PPAC scheme is given in Section 5. Section 6 and Section 7 present the security proof and performance analysis, respectively. Finally, this paper is concluded in Section 8.

## 2. Related Works

Access control is a basic security service in modern computing systems. The access control management ensures that only authorized users are given access to certain resources, which is an effective method to protect data privacy. It is characterized by different access permissions and level of views, and usually constructed according to hierarchical scheme. In particular, Akl and Taylor [24] first proposed the use of cryptography to implement access control in hierarchical structures in 1983. Crampton et al. [25] introduced a novel cryptographic scheme to execute the enforcement of information flow policies. The advantage of this scheme is that no public information is needed to derive the decryption keys. Moreover, when performing a given policy, this tree-based scheme requires fewer keys compared to existing chain-based approaches. Castiglione et al. [26] not only explored the relationship between all the security concepts in the hierarchical key assignment scheme (HKAS), but also proposed a general architecture for HKAS, which provides security for strong key recovery and gives any HKAS that guarantees security for key recovery. According to the security and privacy of outsourced data, a large number of users must create, share, update and delete it dynamically, Castiglione [27] provided some new results on Akl and Taylor’s scheme [24], for flexible and fine-grained access control to support dynamic updates in cloud environments. Alderman [28] designed a space-efficient KAS based on a binary tree, which eliminates public information as well as imposes logarithmic bounds on the number of derivatives required. This scheme performs better than the existing scheme, reduces the storage requirement of user equipment and logarithmically limits the derivation cost.

In 2005, the idea of ABE was proposed by Sahai and Waters [29], which is a one-to-many encryption mechanism. In this scheme, the users encrypt plaintext message based on the certain access control policy and adopt the attributes to identify user’s identities. Afterwards, ABE is divided into ciphertext-policy ABE (CP-ABE) and key-policy ABE (KP-ABE) depending on whether the access structure is associated with the ciphertexts or the sceret keys, respectively. In 2006, the KP-ABE scheme was proposed by Goyal et al. [30], which supports delegation of private keys and provides flexible access policies that enable fine-grained access control. In 2007, Bethencourt et al. [31] constructed the CP-ABE scheme. Even though the storage server is not trusted, this scheme can keep the data confidentiality. In addition, this method could resist collusion attacks. Based on linear secret sharing schemes, Waters [32] firstly put forward a fully expressed CP-ABE scheme in the standard model. The sender of message can formulate an access policy according to its own attributes and define different access policies for different messages in this scheme. The CP-ABE schemes are more appropriate for access control applications, although both KP-ABE and CP-ABE schemes are able to utilize access policies to encrypt message and achieve access control of data. With the development of research, lots of ABE schemes [6,7,8,9,10,11,12,13,14,15,16,17,33,34,35] have been presented.

For guaranteeing the EHR data’s confidentiality in data storage and transmission process, EHR owners must consider the access control of the EHR data with the aim at ensuring merely authorized users can obtain the important information. In 2009, Ibraimi et al. [6] present a novel CP-ABE scheme for safely managing and sharing the EHR data from an un-trusted web server, which is used to force organizational/patient access control policies and protect the data. In 2010, based on cryptographic constructions, Sun et al. [7] proposed a secure EHR system, which combining the mechanisms for revocation and fine-grained access control, and gives support for patient data secure sharing. In 2011, Akinyele et al. [8] designed a self-protecting EHR scheme employing ABE, the main purpose is that the access control policy may be assigned to each encrypted project. In 2013, Li et al. [9] gave a new secure EHR data sharing scheme in cloud computing, which simplified key management for users by using the multi-authorized ABE technique.

Owing to the sensitivity of health relevant data, offering privacy-preserving of EHR owners and access control of the EHR data is the main challenge in nowadays EHR system. Based on public key encryption with keyword search, Narayan et al. [10] proposed an ABE scheme to provide privacy preservation for EHR management system. An attribute-oriented authentication scheme was proposed by Liang et al. [12], which is able to assist an EHR user to establish social relationships and share health information with other trusted users. Lu et al. [13] introduced the user-centric privacy access control scheme and allowed a medical user to determine who may take part in computing to give assistance to the EHR data processing. Liu et al. [14] proposed the online/offline ABE. EHR owners performed most of the encryption calculations during the offline encryption phase. When the access policy and the EHR data were known during the online encryption phase, EHR owners can quickly integrate information to generate the final ciphertext. Zhou et al. [15] presented two anonymous ABE schemes, which can achieve anonymity for personal EHR. On the basis of ABE, a PPAC scheme in mobile healthcare social networks was proposed by Jiang et al. [16]. In this scheme, they adopt bloom filter to hide attributes and efficiently query attributes before decryption. Yang et al. [17] constructed a new attribute bloom filter for the privacy-preserving CP-ABE scheme.

Combining the encryption and digital signature functions in a single step, Zheng [36] firstly proposed the concept of signcryption. And its advantages include that the communication overhead is much smaller than the steps of encryption and signature and it can achieve both confidentiality and authenticity. Combining the idea of ABE and signcryption, attribute-based signcryption (ABSC) has been put forward [18,20,21,22,23,37,38,39,40,41,42,43,44]. In 2010, Gagné et al. [18] proposed the ABSC scheme using the threshold access policy. In which, the users have to determine their access structure in advance in setup phase. In 2011, Wang et al. [20] put forward a ciphertext-policy and claim-predicate ABSC scheme based on bilinear pairings. Its efficiency is much higher than that of the combination of the cipertext policy attribute-based signature (CP-ABS) and CP-ABE. In 2012, the dynamic CP-ABSC scheme was proposed by Emura et al. [21], which allows the signature access structure updating without re-sending the user’s signature key. This is the public verifiability, which permits any intermediary to check the validity of ciphertext before sending it to recipient. In 2013, a novel and security fuzzy attribute-based signcryption scheme was constructed by Hu et al. [22], which enables data encryption, access control, and digital signature for patient medical information in the body area networking. Afterward, based on the bilinear pairings on elliptic curves, Guo et al. [38] realized the concept of ring signcryption in the attribute-based encryption frame and present attributed-based ring singcryption scheme. Wang et al. [39] point that the ABSC scheme [18] is not secure under certain forgery. Han et al. [40] used the inner-product encryption and constructed a threshold ABSC scheme with constant-size ciphertext. In 2014, Wei et al. [41] designed a traceable ABSC scheme. This scheme’s advantage is that the authority could breach anonymity of the signcryption while it is required to trace messages. In 2016, in the light of expressive LSSS access structure, Rao et al. [43] presented an efficient and constant-size ciphertext KP-ABSC scheme. To solve the problem of secure sharing fine-grained access control of the personal health records (PHR) data, Liu et al. [44] proposed a CP-ABSC scheme. Unfortunately, Rao et al. [23] pointed out the problems in scheme [44] and proposed a secure CP-ABSC scheme for the EHR data sharing in cloud.

In summary, the above mentioned ABSC schemes provide the confidentiality and unforegability of the EHR data. However, these schemes cannot specifically solve the problem about the privacy leakage of EHR owners in EHR system. Moreover, the access policies are still in the form of plaintext in these schemes. To a certain extent, the disclosure of the personal privacy information is still a challenging problem in the fine-grained data access control for EHR system.

Besides, now there are many cloud servers supporting two-factor authentication technology. Based on the analysis of the shortcomings of existing two-factor authentication schemes for privacy preserving, Wang et al. [45] proposed an efficient and provably secure two-factor authentication scheme in the random oracle model, which can achieve higher security and privacy without increasing communication or computing costs. In the following study, Wang et al. [46] proposed a two-factor authentication scheme in the random oracle model, which achieves security guarantees beyond the conventional optimal security bound. If an attribute-based authenticated key agreement scheme is constructed on the basis of signcryption technology, it can also provide good security and efficiency in PPAC scheme. In our research, we prefer to design a PPAC solution for EHR system under the standard model. Therefore, in this paper, using the CP-ABSC scheme, we will present the PPAC scheme for the practical and secure EHR system, which prevent the leakage of EHR owner’s personal privacy information from the access policy and may achieve fine-grained access control of EHR data.

## 3. Preliminaries

### 3.1. Bilinear Pairings

Let G, $G_{T}$ be two multiplicative cyclic groups of prime order *p* and *g* be the generator of G. The bilinear map $e:G\times G\to G_{T}$ satisfies the following three properties:

1. Bilinearity: For all u,v∈G and $a,b\in Z_{p}$, where $e\left(u^{a},v^{b}\right)=e{\left(u,v\right)}^{ab}$.
2. Non-degeneracy: e(g,g)≠1.
3. Computability: For all u,v∈G, there exists an efficient algorithm to compute e(u,v) for all u,v∈G.

### 3.2. Access Structures

Suppose $P=\{P_{1},P_{2},\cdots ,P_{n}\}$ is a set of parties. There exists a collection $W\subseteq 2^{P}$, which is monotone if and only if for any set *B* and *C*, if B∈W and B⊆C then C∈W. An access structure is a collection *W* of non-empty subsets of $\left\{P_{1},P_{2},\cdots ,P_{n}\right\}$, i.e., $W\subseteq 2^{P}\setminus \left\{\emptyset \right\}$. The sets in *W* are named as the authorized sets, otherwise which are named as the unauthorized sets.

### 3.3. Linear Secret Sharing Schemes

A secret sharing scheme Π for access structure *W* is called the linear secret sharing scheme (LSSS) over a set of parties *P* in $Z_{p}$ if

1. The shares for each party form a vector over $Z_{p}$.
2. There exists a share-generating matrix *M* with *l* rows and *n* columns for Π. For all i=[1,l], ρ(i) maps the *i*’th row of *M* to every authorized role attribute, where the function ρ is a function from {1,2,⋯,l} to *P*. We find a column vector $\vec{v}=\left(\sigma ,r_{2},\cdots ,r_{n}\right)$ be a sharing vector, where $r_{2},\cdots ,r_{n}\in Z_{p}$ are random values and $\sigma \in Z_{p}$ is the secret value to be shared. $M\vec{v}$ is the vector of *l* shares of σ on Π. Each $\lambda_{i}={\left(M\vec{v}\right)}_{i}$ is distributed as secret share value to each attribute ρ(i).

**Figure 1:**
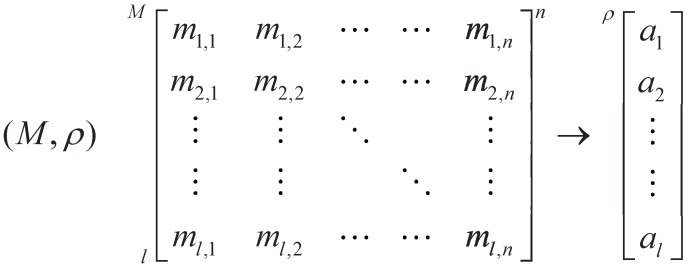
The LSSS access policy.

An LSSS to be represented by an access structure W=(M,ρ) is shown in Figure 1. Each LSSS has the linear reconstruction property, defined as follows: Let *W* be the access structure and Π be the LSSS. For any authorized set, i.e., S∈W, let I={i:ρ(i)∈S}⊂{1,2,⋯,l}. According to Π, if ${\left\{\lambda_{i}\right\}}_{i\in I}$ are valid shares for the secret σ, here exists constants ${\left\{w_{i}\in Z_{p}\right\}}_{i\in I}$ such that $\sum_{i\in I}w_{i}\lambda_{i}=\sigma$. Let $M_{i}$ denote *i*’th row of *M*, then $\sum_{i\in I}w_{i}M_{i}=\left(1,0,\cdots ,0\right)$. It is worth noting that the constants $\left\{w_{i}\right\}$ can be obtained in time polynomial in scale of the share-generation matrix *M*.

### 3.4. Cuckoo Filter

The data structure called cuckoo filter [19] is the extended version of bloom filter, which supports adding and removing items dynamically while having lower space overhead, shorter search time and better performance than bloom filter [47]. It also solves the problem of false positive in bloom filter. As a method for testing set membership, cuckoo filter uses cuckoo hashing technique [48] to solve the problem of false positive in bloom filter and check whether an element exists in a set.

**Figure 2:**
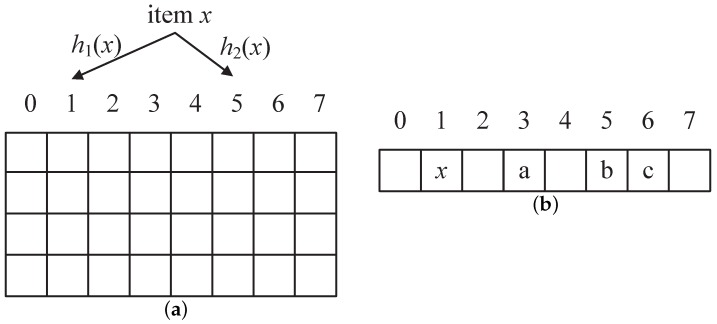
Cuckoo hashing table. (**a**) the basic cuckoo hashing table; (**b**) inserting a new element.

Figure 2a shows the basic cuckoo hashing table that includes a series of buckets, and each bucket contains 4 entries. There are two candidate buckets in every item *x*, which are calculated from the formula and $h_{1}\left(x\right)$ and $h_{2}\left(x\right)$. The process of inserting a new element into the hash table is displayed as Figure 2b. In Figure 2, the hash table has 8 buckets. When adding a new element into the candidate bucket 1 or 5, if either of the two candidate buckets is empty, we will insert it into the other free bucket.If both buckets have no space the element selects any candidate bucket (such as “1”) and removes the existing element, then this moved element need to re-insert into itself alternative position as shown in Figure 2b. In this case, it will trigger the item “c” that removes from bucket 3 into bucket 6 when removing “a”. We will repeat this operation until we find an empty bucket and the maximum number of times is reached. When no empty bucket is obtained, the cuckoo hashing table will be regard as that it is too filled to insert.

A cuckoo filter algorithm has mainly three functions: the insert function that stores items into the filter, the lookup function that checks whether an item exists in the filter and the delete function that removes the previously inserted items. For each item *x*, cuckoo filter stores a fingerprint and calculates two candidate buckets $i_{1}$ and $i_{2}$ by the following formulas:

(1) $$i_{1}=H_{4}\;\left(x\right)$$

(2) $$i_{2}=i_{1}⊕H_{4}\;\;\left(fingerprin\;\;t\left(x\right)\right)$$

where $H_{4}$ is a one-way hash function.

We only adopt the insert and lookup functions of cuckoo filter in our paper. Algorithm 1 and Algorithm 2 illustrate the insert operation and lookup operation, respectively.

In Algorithm 1, cuckoo filter adds new items dynamically through storing fingerprints *f* of every item *x*. In Algorithm 2, we can easily check whether an item *y* belong to cuckoo filter.

**Algorithm 1** Insert (x)f=fingerprint(x); $i_{1}=H_{4}\left(x\right)$; $i_{2}=i_{1}⊕H_{4}\left(x\right)$; **If** bucket $\left[i_{1}\right]$ or bucket $\left[i_{2}\right]$ has an empty entry **then** add *f* to that bucket; return Done; *i* = randomly pick $i_{1}$ or $i_{2}$; **For**
n=0; n<MaxMumKicks; n++
**do** randomly select an entry *e* from bucket [i]; swap *f* and fingerprint stored in entry *e*; $i=i⊕H_{4}\left(f\right)$; **If** bucket [i] has an empty entry **then** add *f* to bucket [i]; **return** done; **return** False.

**Algorithm 2** Lookup (y)f=fingerprint(y); $i_{1}=H_{4}\left(x\right)$; $i_{2}=i_{1}⊕H_{4}\left(x\right)$; **If** bucket $\left[i_{1}\right]$ or bucket $\left[i_{2}\right]$ has *f*
**then** **return** True; **else** **return** False; **End If**.

### 3.5. Complexity Assumptions

Decisional *q*-Bilinear Diffie-Hellman Exponent (*q*-DBDHE) Problem: Given the tuple $y_{a,\sigma }=\left(g,g^{\sigma },g^{a},g^{a^{2}},\cdots ,g^{a^{q}},g^{a^{q+2}},\cdots ,g^{a^{2q}}\right)$ in group G and $a,\sigma \in Z_{p}$ are chosen at randomly, the task of *q*-DBDHE problem is to distinguish $e\left(g^{a^{q+1}},g^{\sigma }\right)\in G_{T}$ from a random element $R\in G_{T}$.

The advantage of A in solving the *q*-DBDHE problem is defined as

$$Adv_{A}^{q-\mathrm{DBDHE}}=\mathrm{Pr}\left[1\leftarrow A\left(y_{a,\sigma },T\right)|T=e{\left(g,g\right)}^{a^{q+1}\sigma }\right]-\mathrm{Pr}\left[1\leftarrow A(y_{a,\sigma },T)|T=R\right]\geq \varepsilon .$$

*q*-DBDHE Assumption: It says that there is no known polynomial-time algorithm A to solve the *q*-DBDHE problem with advantage at least ε.

Computational *q*-Diffie-Hellman Exponent (*q*-CDHE) Problem: Given the tuple $y_{a}=\left(g,g^{a},g^{a^{2}},\cdots ,g^{a^{q}},g^{a^{q+2}},\cdots ,g^{a^{2q}}\right)$ in group G and $a\in Z_{p}$ is chosen at randomly, the task of *q*-CDHE problem is to compute $g^{a^{q+1}}$.

The advantage of in solving the *q*-CDHE problem is defined as

$$Adv_{A}^{q-\mathrm{CDHE}}=\mathrm{Pr}\left[g^{a^{q+1}}\leftarrow A\left(y_{a}\right)\right]\geq \varepsilon .$$

*q*-CDHE Assumption: It says that there is no known polynomial-time algorithm A to solve the *q*-CDHE problem with advantage at least ε.

## 4. Model

In this section, we first give the typical structure of the EHR system model and the specific working stages of the proposed PPAC scheme for the EHR system model. Then, we define a CP-ABSC scheme and its security model, which is the basic method to implement the proposed PPAC scheme.

### 4.1. System Model

**Figure 3:**
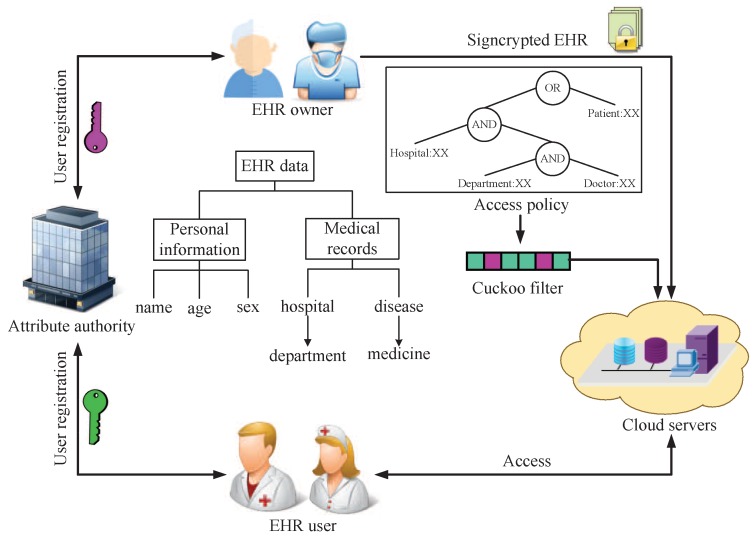
A framework of the EHR system.

A typical structure of EHR system model is demonstrated in Figure 3.

EHR system comprises four entities: Attribute authority (AA), EHR owner, EHR user and Cloud servers.

- AA is a trusted party that is responsible for generating and distributing public parameters and private keys for the users, selects attributes from the attribute space and assigns to the users with different rights.
- EHR owner is the EHR data provider (such as a patient) who formulates the access policy, signcrypts his/her own EHR data and uploads the ciphertext to cloud servers.
- EHR user is the EHR data receiver (such as a doctor or nurse) who can download the cipgertext from cloud servers and unsigncrypt it.
- Cloud servers are in charge of storing ciphertext data that sent by the EHR owner and granting access rights to EHR users.

On the basis of the above EHR system model, our paper designs a new PPAC scheme for the EHR system, which includes the following four phases.

- **System initialization phase**: AA generates the master key and public systems parameters for EHR system, and then publishes the system parameters to all users (EHR owners and EHR users).
- **Users registration phase**: The users submit a registration application to AA. AA verifies the legitimacy of the identity of the user according to the attributes owned by itself and distributes corresponding private key to the user.
- **EHR signcrypt phase**: An EHR owner signcrypts the EHR data (such as personal information and medical records) under the access policy, hides the access policy by the cuckoo filter and uploads the ciphertext to cloud servers for data sharing.
- **EHR access phase**: An EHR user submits the data access request to the cloud servers, who can download ciphertext from cloud servers and unsigncrypt data to obtain original messages if and only if the attribute set of EHR user that satisfies access policy.

### 4.2. Security Model

The CP-ABSC scheme is composed of the following five algorithms [23,29]:

**Setup**: Given a security parameter *k*, system attribute set *S* and message universe M, the algorithm outputs the master key *MSK* and system public parameters *PK*.

**sExtract**: Given *PK*, *MSK* and the signing attribute set $A_{s}\subseteq S$, the algorithm outputs the corresponding signing private key $SK_{A_{s}}$.

**dExtract**: Given *PK*, *MSK* and the decryption attribute set $A_{d}\subseteq S$, the algorithm outputs the corresponding decryption private key $SK_{A_{d}}$.

**Signcrypt**: Given *PK*, the message m∈M, the signing private key $SK_{A_{s}}$ for $A_{s}$, the encryption access structure $W_{e}=\left(M_{e},\rho_{e}\right)$, signing access structure $W_{s}=\left(M_{s},\rho_{s}\right)$, where $A_{s}\in W_{s}$, and the cuckoo filter, the algorithm outputs the ciphertext *CT*.

**Unsigncrypt**: Given *PK*, the ciphertext *CT* and the decryption private key $SK_{A_{d}}$ for $A_{d}$, the algorithm firstly queries the corresponding attributes values by cuckoo filter and reconstructs the access structure $W_{e}^{\prime }=\left(M_{e},\rho_{e}^{\prime }\right)$, and outputs message *m* if $A_{d}\in W_{e}^{\prime }$. Otherwise, the algorithm returns ⊥.

According to [23,32], the security of CP-ABSC needs to satisfy confidentiality and unforgeability.

The confidentiality (indistinguishability against adaptive chosen ciphertext attack (IND-CCA2)) for CP-ABSC is captured by an interactive game between the adversary A and the challenger C as follows.

**Initialization**: The adversary A chooses an encryption access structure $W_{e}^{*}$ for the encryption attribute set $A_{d}$, which is applied to calculate the challenge ciphertext and provides it to the challenger C.

**Setup**: C executes the **Setup** algorithm. C keeps the master key *MSK* secretly and returns the public parameters *PK* to A.

**Phase 1**: A adaptively issues the following polynomial bounded queries.

- **sExtract queries**: Given a query on the signing attribute set $A_{s}$, C executes the **sExtract** algorithm and returns the corresponding private key $SK_{A_{s}}$ to A.
- **dExtract queries**: Given a query on the decryption attribute set $A_{d}\notin W_{e}^{*}$, C executes the **dExtract** algorithm and returns the corresponding private key $SK_{A_{d}}$ to A.
- **Signcrypt queries**: Given a query on the message m∈M, the decryption attribute set $A_{d}$, the signing attribute set $A_{s}$, the encryption access structure $W_{e}$, the signing access structure $W_{s}$ and cuckoo filter, C executes the **sExtract** algorithm and obtains the signing private key $SK_{A_{s}}$. Then C execute the **Signcrypt** algorithm to generate the ciphertext *CT* and returns to A.
- **Unsigncrypt queries**: Given a query on the ciphertext *CT*, the decryption attribute set $A_{d}$ and the signing attribute set $A_{s}$, C firstly queries the corresponding attributes of EHR users that are in cuckoo filter or not and reconstructs the access structure $W_{e}^{\prime }=\left(M_{e},\rho_{e}^{\prime }\right)$. C executes the **dExtract** algorithm and obtains the decryption private key $SK_{A_{d}}$. And C executes the **Unsigncrypt** algorithm to obtain the message *m* and returns to A.

**Challenge**: After completing the Phase 1, A outputs two equal length messages $m_{0}^{*},m_{1}^{*}$ and the signing access structure $W_{s}^{*}$. When the signing attribute set $A_{s}^{*}\in W_{s}^{*}$, C gets $SK_{A_{d}^{*}}$ by running the **dExtract** algorithm. C randomly chooses θ∈{0,1} and executes the **Signcrypt** algorithm to generate the ciphertext $CT^{*}$. At last, C sends $CT^{*}$ to A as its challenge ciphertext.

**Phase 2**: A adaptively issues the queries as in Phase 1 except the **dExtract queries** for any decryption attribute set $A_{d}\in W_{e}^{*}$ and the **Unsigncrypt queries** for the challenge ciphertext $CT^{*}$ for any $A_{d}\in W_{e}^{*}$.

**Guess**: A outputs a guess bit θ∈{0,1}. If $\theta^{\prime }=\theta$, A wins the above game.

The advantage of A that wins the above game is defined to be $Adv=|\mathrm{Pr}\left[\theta^{\prime }=\theta \right]-\frac{1}{2}|$.

**Definition 1(Confidentiality)**. A CP-ABSC scheme is IND-CCA2 security, if there is no polynomial-time adversary who wins the aforementioned game with the non-negligible advantage.

The unforgeability (existential unforgeability against adaptive chosen message attack (EUF-CMA)) for CP-ABSC is captured by an interactive game between the adversary A and the challenger C as follows.

**Initialization**: The adversary A provides the challenge signing access structure $W_{s}^{*}$ to the challenger C.

**Setup**: C executes the **Setup** algorithm. Then C keeps the master key *MSK* secretly and returns the public parameters *PK* to A.

**Query phase**: A performs a polynomial bounded number of queries adaptively.

- **sExtract queries**: Give a query on the signing attributes set $A_{s}\notin W_{s}^{*}$, C executes the **sExtract** algorithm and returns the corresponding private key $SK_{A_{s}}$ to A.
- **dExtract queries**: Give a query on the decryption attributes set $A_{d}$, C executes the **sExtract** algorithm and returns the corresponding private key $SK_{A_{d}}$ to A.
- **Signcrypt queries**: Same as the **Signcrypt queries** in the confidentiality game.
- **Unsigncrypt queries**: Same as the **Unsigncrypt queries** in the confidentiality game.

**Forgery**: A outputs the forgery ciphertext $CT^{*}$ on $\left(m^{*},W_{s}^{*},W_{e}^{*}\right)$.

A wins above game if $CT^{*}$ is valid and A never makes the **Signcrypt queries** on $\left(m^{*},W_{s}^{*},W_{e}^{*}\right)$.

The advantage of A that wins the above game is defined as the probability that it wins the unforgeability game.

**Definition 2(Unforgeability)**. A CP-ABSC scheme is EUF-CMA security, if there is no polynomial-time adversary who wins the aforementioned game with the non-negligible advantage.

## 5. The Proposed Scheme

The construction of PPAC scheme for EHR system is based on the CP-ABSC scheme and the concrete CP-ABSC scheme is given based on the bilinear pairing, supporting the linear secret sharing schemes. Employing the cuckoo filter to hide the access policy, it could protect the EHR owner’s privacy information. The proposed scheme meets the requirements of PPAC in this section, by using CP-ABSC mechanism to signcrypt plaintext messages can satisfy the confidentiality and unforegability of the EHR data. At the same time, the use of cuckoo filter achieves the purpose of privacy preserving. Specifically, our proposed CP-ABSC scheme includes four phases: system initialization, user registration phase, EHR signcrypt phase and EHR access phase. The detail steps are as follows.

### 5.1. System Initialization

AA generates the master key *MSK* and public parameters *PK* for EHR system through executing the **Setup** algorithm.

- **Setup**: Given the security parameter *k*, message universe $M:{\left\{0,1\right\}}^{*}$ and attribute set *S* that includes the EHR owner’s attributes and EHR user’s attributes. AA picks three collision resistant cryptographic hash functions: $H_{1}:{\left\{0,1\right\}}^{*}\to {\left\{0,1\right\}}^{l}$, $H_{2}:G\to Z_{p}^{*}$, $H_{3}:\left\{0,1\right\}\to Z_{p}^{*}$. Besides, AA chooses a one-way hash function $H_{4}:\left\{0,1\right\}\to Z_{p}^{*}$, which will be used to hash all ρ(i) for i∈{1,2,⋯,l} in the access policy W=(M,ρ) associated with the EHR owners’ attributes. Then, AA randomly chooses $a,\alpha \in Z_{p}^{*}$, $\delta_{1},\delta_{2},y_{0},y_{1},\cdots ,y_{l}\in G$ and sets $Y=e{\left(g,g\right)}^{\alpha }$. For each attribute x∈S, AA samples $h_{x}\in G$.

The system parameters are $PK=\{M,S,H_{1},H_{2},H_{3},H_{4},g^{a},\delta_{1},\delta_{2},y_{0},{\left\{y_{i}\right\}}_{i\in [1,l]},Y,{\left\{h_{x}\right\}}_{x\in S}\}$ and the master key is $MSK=\{g^{\alpha }\}$.

### 5.2. User Registration Phase

According to the attributes of the EHR owner and the EHR user, AA generates the corresponding private keys through executing the **sExtract** and **dExtract** algorithms.

- **sExtract**: Given *PK*, *MSK* and the signing attribute set $A_{s}\subseteq S$, AA randomly selects $r_{s}\in Z_{p}^{*}$ and outputs the EHR owner’s signing private key $SK_{A_{s}}:K_{s}=g^{\alpha }g^{ar_{s}}$, $L_{s}=g^{r_{s}}$, ${\left\{K_{s,x}=h_{x}^{r_{s}}\right\}}_{x\in A_{s}}$.
- **dExtract**: Given *PK*, *MSK* and the decryption attribute set $A_{d}\subseteq S$, AA randomly picks $r_{d}\in Z_{p}^{*}$ and outputs the EHR user’s decryption private key $SK_{A_{d}}:K_{d}=g^{\alpha }g^{ar_{d}}$, $L_{d}=g^{r_{d}}$, ${\left\{K_{d,x}=h_{x}^{r_{d}}\right\}}_{x\in A_{d}}$.

### 5.3. EHR Signcrypt Phase

The EHR owner signcrypts his/her own EHR data and uses cuckoo filter to hide the access policy *W* associated with attributes through executing the **Signcrypt** algorithm.

- **Signcrypt**: Given the message m∈M, the signing private key $SK_{A_{s}}$, and the encryption access policy $W_{e}=\left(M_{e},\rho_{e}\right)$ and the signing access policy $W_{s}=\left(M_{s},\rho_{s}\right)$ that are formulated by the EHR owner. The EHR owner performs the following steps.
  - The EHR owner selects a vector $\vec{v}=\left(\sigma ,v_{2},\cdots ,v_{n}\right)\in Z_{p}^{*}$ calculates $\lambda_{i}=\vec{v}\cdot M_{i}$ for i=1,2,⋯,l, where $M_{i}$ is the *i*’th row of matrix *M*. And the EHR owner randomly chooses $\varphi_{i}\in Z_{p}$ and generators a vector $\vec{\varphi }=\left(-\varphi_{1},-\varphi_{2},\cdots ,-\varphi_{l}\right)$ such that $\vec{\varphi }\cdot M_{s}=-{\vec{1}}_{n}$, that is $\sum_{i=1}^{l}\varphi_{i}\cdot M_{s,i}=-{\vec{1}}_{n}$, and $\varphi_{i}=0$ for all *i* where $\rho_{s}\left(i\right)\notin A_{s}$, where $M_{s,i}$ is the *i*’th row of matrix $M_{s}$.
  - The EHR owner picks $\xi \in Z_{p}^{*}$ and computes
    $$C=mY^{\sigma },C^{\prime }=g^{\sigma },\mu =H_{2}\left(C^{\prime }\right),C^{\prime \prime }={\left({\delta_{1}}^{\mu }\delta_{2}\right)}^{\sigma },\left\{C_{i}=g^{a\lambda_{i}}h_{\rho_{e}\left(i\right)}^{-\sigma }\right\}{\;}_{i\in [1,l]},$$
    $$S_{1}=L_{s}=g^{r_{s}},H_{1}\left(S_{1},W_{e},W_{s}\right)=\left(j_{1},j_{2},\cdots ,j_{l}\right),$$
    $$H_{3}\left(W_{e},W_{s},C,C^{\prime },C^{\prime \prime },\left\{C_{i}=g^{a\lambda_{i}}h_{\rho_{e}\left(i\right)}^{-\sigma }\right\}{\;}_{i\in [1,l]}\right)=\beta$$
    $$S_{2}=K_{s}\cdot \prod_{i\in [1,l]}{\left(K_{s,\rho_{s}}\right)}^{\varphi_{i}}\cdot {\left(y_{0}\prod_{i\in [1,l]}{y_{i}}^{j_{i}}\right)}^{\sigma }\cdot {\left(C^{\prime \prime }\right)}^{\beta \xi }.$$
  - The EHR owner uses the cuckoo filter to hide the access policy $W_{e}=\left(M_{e},\rho_{e}\right)$. In order to derive the alternative position of an item based on its fingerprint, it needs to utilize the partial-key cuckoo hashing [19]. That can ensure the EHR owner inserts new items to cuckoo filter dynamically. For each valid attribute $a_{i}\in S$, where the attribute $a_{i}=\rho_{e}\left(i\right)$ maps the *i*’th row of access matrix *M*, let item $x=a_{i}$. The EHR owner dynamically inserts a new item *x* into the cuckoo filter by using the insert operation as shown in Algorithm 1 and constructs the cuckoo filter data structure *CF*. Finally, the EHR owner uploads the ciphertext $CT=\{C,C^{\prime },C^{\prime \prime },{\left\{C_{i}\right\}}_{i\in [1,l]},S_{1},S_{2},CF\}$ to the cloud server.

### 5.4. EHR Access Phase

In this phase, the EHR user downloads the ciphertext *CT* from the cloud servers, then gets message *m* through running the **Unsigncrypt** algorithm.

- **Unsigncrypt**: Given the ciphertext *CT*, the EHR user performs the following steps.
  - Suppose that $S^{\prime }$ is the attribute set of the EHR user. For every attribute $a_{i}^{\prime }\in S^{\prime }$, let an item $y=a_{i}^{\prime }$. The EHR user first checks the attributes are in the access policy or not by using using the lookup operation of the cuckoo filter as shown in Algorithm 2. If the item *y* is in cuckoo filter, it means that the attribute $a_{i}^{\prime }$ exists in the access policy. Lastly, the EHR user generates the reconstructed attribute map $\rho_{e}^{\prime }\left(i\right)=a_{i}^{\prime }$ and obtains the access policy $W_{e}^{\prime }=\left(M_{e},\rho_{e}^{\prime }\right)$.
  - The EHR user computes $\mu =H_{2}\left(C^{\prime }\right)$, $H_{1}\left(S_{1},W_{e}^{\prime },W_{s}\right)=\left(j_{1},j_{2},\cdots ,j_{l}\right)$, $\beta =H_{3}\left(W_{e}^{\prime },W_{s},C,C^{\prime },C^{\prime \prime },{\left\{C_{i}=g^{a\lambda_{i}}h_{\rho_{e}\left(i\right)}^{-\sigma }\right\}}_{i\in [1,l]}\right)$ and verifies
    (3) $$Y=\frac{e(S_{2},g)}{e\left(g^{a}\cdot \prod_{i\in [1,l]}h_{\rho_{s}\left(i\right)}^{\varphi_{i}},S_{1}\right)\cdot e\left(y_{0}\prod_{i\in [1,l]}{y_{i}}^{j_{i}}\cdot {\left({\delta_{1}}^{\mu }\delta_{2}\right)}^{\beta \xi },C^{\prime }\right)}$$
  - If it is invalid, returns ⊥; Otherwise, when the decryption attribute set $A_{d}\in S^{\prime }$ satisfies $\left(M_{e},\rho_{e}^{\prime }\right)$, the EHR user finds the constants ${\left\{\omega_{i}\in Z_{p}^{*}\right\}}_{i\in I}$ such that $\left\{\lambda_{i}\right\}$ are valid shares of secret value σ based on $M_{e}$, $\sum_{i\in I}\omega_{i}\lambda_{i}=\sigma$, where $I=\{i:\rho_{e}^{\prime }\left(i\right)\in S^{\prime }\}$.
    The EHR user computes
    (4) $$Y^{\sigma }=\frac{e(C^{\prime },K_{d})}{\prod_{i\in I}{\left(e\left(C_{i},L_{d}\right)\cdot \;e\left(C^{\prime },K_{d,{\rho^{\prime }}_{e}}\right)\right)}^{\omega_{i}}}$$
    and recovers the message *m* from $m=\frac{C}{Y^{\sigma }}$.

**Correctness**:

$$\begin{array}{cc} S_{2} & =K_{s}\cdot \prod_{i\in [1,l]}{\left(K_{s,\rho_{s}}\right)}^{\varphi_{i}}\cdot {\left(y_{0}\prod_{i\in [1,l]}{y_{i}}^{j_{i}}\right)}^{\sigma }\cdot {\left(C^{\prime \prime }\right)}^{\beta \xi }\\ & =g^{\alpha }g^{ar_{s}}\cdot \prod_{i\in [1,l]}{\left(h_{\rho_{s}\left(i\right)}^{r_{s}}\right)}^{\varphi_{i}}\cdot {\left(y_{0}\prod_{i\in [1,l]}{y_{i}}^{j_{i}}\right)}^{\sigma }\cdot {\left(C^{\prime \prime }\right)}^{\beta \xi }, \end{array}$$

$$\begin{array}{cc} e(S_{2},g) & =e\left(g^{\alpha }g^{ar_{s}},g\right)\cdot e\left(\prod_{i\in [1,l]}{\left(h_{\rho_{s}\left(i\right)}^{r_{s}}\right)}^{\varphi_{i}},g\right)\cdot e\left({\left(y_{0}\prod_{i\in [1,l]}{y_{i}}^{j_{i}}\right)}^{\sigma },g\right)\cdot e\left({\left(\delta_{1}^{\mu }\delta_{2}\right)}^{\sigma \beta \xi },g\right)\\ & =e{\left(g,g\right)}^{\alpha }\cdot e\left(g^{a},g^{r_{s}}\right)\cdot e\left(\prod_{i\in [1,l]}{\left(h_{\rho_{s}\left(i\right)}\right)}^{\varphi_{i}},g^{r_{s}}\right)\cdot e\left(y_{0}\prod_{i\in [1,l]}y_{i}^{j_{i}},g^{\sigma }\right)\cdot e\left({\left(\delta_{1}^{\mu }\delta_{2}\right)}^{\beta \xi },g^{\sigma }\right)\\ & =Y\cdot e\left(g^{a}\prod_{i\in [1,l]}h_{\rho_{s}\left(i\right)}^{\varphi_{i}},S_{1}\right)\cdot e\left(y_{0}\prod_{i\in [1,l]}y_{i}^{j_{i}}\cdot {\left(\delta_{1}^{\mu }\delta_{2}\right)}^{\beta \xi },C^{\prime }\right), \end{array}$$

$$\begin{array}{cc} \frac{e(C^{\prime },K_{d})}{\prod_{i\in I}{\left(e\left(C_{i},L_{d}\right)\cdot \;e\left(C^{\prime },K_{d,{\rho^{\prime }}_{e}}\right)\right)}^{\omega_{i}}} & =\frac{e(g^{\sigma },g^{\alpha }g^{ar_{d}})}{\prod_{i\in I}{\left(e\left(g^{a\lambda_{i}}h_{{\rho^{\prime }}_{e}\left(i\right)}^{-\sigma },g^{r_{d}}\right)\cdot e\left(g^{\sigma },h_{{\rho_{e}}^{\prime }\left(i\right)}^{r_{d}}\right)\right)}^{\omega_{i}}}\\ & =\frac{e{\left(g,g\right)}^{\alpha \sigma }\cdot e{\left(g,g\right)}^{a\sigma r_{d}}}{\prod_{i\in I}e{\left(g,g\right)}^{ar_{d}\lambda_{i}\omega_{i}}}=e{\left(g,g\right)}^{\alpha \sigma }=Y^{\sigma }. \end{array}$$

## 6. Security Proof

### 6.1. Confidentiality

**Theorem** **1.** *Assuming there is the adversary A who is capable of breaking the IND-CCA2 security of CP-ABSC scheme with a non-negligible probability ε, then we we can construct an algorithm B that solves the *q*-DBDHE problem with the probability at least $\varepsilon^{\prime }=\varepsilon -\frac{q_{us}}{p}$, where $q_{us}$ is the maximum number of the **Unsigncrypt queries** issued by A.*

**Proof.**  The algorithm B receives an instance $y_{a,\sigma }=\left(g,g^{\sigma },g^{a},g^{a^{2}},\cdots ,g^{a^{q}},g^{a^{q+2}},\cdots ,g^{a^{2q}}\right)$ of the *q*-DBDHE problem, where $g_{i}=g^{a^{i}}$, $a,\sigma \in Z_{p}$ and *g* is a generator of G. The goal of B is to decide whether $T=e{\left(g,g\right)}^{a^{q+1}\sigma }$ or T=R, where *R* is a random element in $G_{T}$. If $T=e{\left(g,g\right)}^{a^{q+1}\sigma }$, B outputs 1; Otherwise outputs 0. Then B chooses three collision-resistant hash functions $H_{1}:{\left\{0,1\right\}}^{*}\to {\left\{0,1\right\}}^{l}$, $H_{2}:G\to Z_{p}^{*}$, $H_{3}:\left\{0,1\right\}\to Z_{p}^{*}$ and a one-way hash function $H_{3}:\left\{0,1\right\}\to Z_{p}^{*}$. The algorithm B simulates the challenger in IND-CCA2 security game and interacts with the adversary A as below. □

**Initialization**: A submits the message space $M:{\left\{0,1\right\}}^{*}$ and the challenge encryption access structure $W_{e}^{*}=\left(M_{e}^{*},\rho_{e}^{*}\right)$ to B, where $M_{e}^{*}$ is a matrix of $l^{*}\times n^{*}$ with the labeling function $\rho_{e}^{*}$. Let ${\vec{M}}_{i}^{*}=\left(M_{i,1}^{*},M_{i,2}^{*},\cdots ,M_{i,n^{*}}^{*}\right)$ be the *i*’th row of $M_{e}^{*}$.

**Setup**: B chooses a random $\alpha^{\prime }\in Z_{p}^{*}$ and calculates $\alpha =\alpha^{\prime }+a^{q+1}$, $Y=e\;\;{\left(g,g\right)}^{\alpha }=e\;\left(g^{a},g^{a^{q}}\right)\cdot e\;\;{\left(g,g\right)}^{\alpha^{\prime }}$. B randomly chooses $\varsigma \in Z_{p}^{*}$, $\eta_{0},\eta_{1},\cdots ,\eta_{l}\in Z_{p}^{*}$ and sets $C^{\prime *}=g^{\sigma }$, $\mu^{*}=H_{2}\left(C^{\prime *}\right)$, $\delta_{1}=g_{q}^{\frac{1}{\mu^{*}}}$, $\delta_{2}=g^{\varsigma }g_{q}^{-1}$, $y_{0}=g^{\eta_{0}}$, $y_{1}=g^{\eta_{1}},\cdots ,y_{l}=g^{\eta_{l}}$.

Finally, for each attribute x∈S, let *X* denote the set of indices *i* such that $\rho_{e}^{*}\left(i\right)=x$. If X≠∅, B selects a random parameter $f_{x}\in Z_{p}^{*}$ and defines $h_{x}=g^{f_{x}}\cdot g^{aM_{i,1}^{*}}\cdot g^{a^{2}M_{i,2}^{*}}\cdots g^{a^{n^{*}}M_{i,n}^{*}}$. If X=∅, then $h_{x}=g^{f_{x}}$.

B returns the public parameters $PK=\{S,M,H_{1},H_{2},H_{3},H_{4},Y,\delta_{1},\delta_{2},y_{0},{\left\{y_{i}\right\}}_{i\in [1,l]},Y,{\left\{h_{x}\right\}}_{x\in S}\}$ to A.

**Phase 1**: A adaptively makes a number of queries as follows.

- **sExtract queries**: When A issues a query on the signing attribute set $A_{s}$, B randomly chooses $\hat{r}\in Z_{p}^{*}$, sets $r_{s}=\hat{r}-a^{q}$ and computes $L_{s}=g^{\hat{r}}g_{q}^{-1}$, $K_{s}=g^{\alpha^{\prime }}g_{1}^{\hat{r}}$, $K_{s,x}=h_{x}^{\hat{r}}g_{q}^{-f_{x}}$ for any $x\in A_{s}$. Then B returns the signing private key $SK_{A_{s}}=\left\{L_{s},K_{s},{\left\{K_{s,x}\right\}}_{x\in A_{s}}\right\}$ to A.
  **Correctness**:
  $$L_{s}=g^{\hat{r}}g_{q}^{-1}=g^{\hat{r}}g^{-a^{q}}=g^{r_{s}},$$
  $$K_{s}=g^{\alpha^{\prime }}g_{1}^{\hat{r}}=g^{\alpha^{\prime }}g_{q+1}g_{1}^{\hat{r}}g_{q+1}^{-1}=g^{\alpha^{\prime }+a^{q+1}}g^{a\hat{r}-a^{q+1}}=g^{\alpha }g^{ar_{s}},$$
  $$K_{s,x}=h_{x}^{\hat{r}}g_{q}^{-f_{x}}=h_{x}^{\hat{r}}{\left(h_{x}\right)}^{-a^{q}}=h_{x}^{r_{s}}.$$
- **dExtract queries**: When A issues a query on the decryption attributes set $A_{d}\notin W_{e}^{*}$, B randomly chooses a vector $\vec{\gamma }=\left(\gamma_{1},\gamma_{2},\cdots ,\gamma_{n^{*}}\right)\in Z_{p}^{n^{*}}$ where $\gamma_{1}=-1$, $\vec{\gamma }\cdot M_{e,i}^{*}=0$ for all *i* where $\rho_{e}^{*}\left(i\right)\in A_{d}$. B randomly selects $\hat{r}\in Z_{p}^{*}$, implicitly defines $r_{d}=\hat{r}+\gamma_{1}a^{q}+\gamma_{2}a^{q-1}+\cdots +\gamma_{n^{*}}a^{q-n^{*}+1}$ and computes $L_{d}=g^{\hat{r}}\prod_{i=1}^{n^{*}}{\left(g^{a^{q+1-i}}\right)}^{\gamma_{i}}$, $K_{d}=g^{\alpha^{\prime }}g^{a\hat{r}}\prod_{i=2}^{n^{*}}{\left(g^{a^{q+2-i}}\right)}^{\gamma_{i}}$ and $K_{d,x}=L_{d}^{f_{x}}\prod_{j=1}^{n^{*}}{\left(g^{a^{j}\cdot \hat{r}}\prod_{\underset{o\neq j}{o=1,\cdots ,n^{*}}}{\left(g^{a^{q+1+j-o}}\right)}^{\gamma_{o}}\right)}^{M_{i,j}^{*}}$ for any $x\in A_{d}$. For any $i\in [1,l_{e}^{*}]$, if there is no $\rho_{e}^{*}\left(i\right)=x$, then B simply sets $K_{d,x}=L_{d}^{f_{x}}$. Then B returns the decryption key $SK_{A_{d}}=\left\{L_{d},K_{d},{\left\{K_{d,x}\right\}}_{x\in A_{d}}\right\}$.
  **Correctness**:
  $$L_{d}=g^{\hat{r}}\prod_{i=1}^{n^{*}}{\left(g^{a^{q+1-i}}\right)}^{\gamma_{i}}=g^{r_{d}},$$
  $$\begin{array}{cc} K_{d} & =g^{\alpha^{\prime }}g^{a\hat{r}}\prod_{i=2}^{n^{*}}{\left(g^{a^{q+2-i}}\right)}^{\gamma_{i}}=g^{\alpha^{\prime }}g^{a^{q+1}}\cdot g^{a\hat{r}}\cdot g^{-a^{q+1}}\prod_{i=2}^{n^{*}}{\left(g^{a^{q+2-i}}\right)}^{\gamma_{i}}=g^{\alpha }g^{a\hat{r}}\prod_{i=1}^{n^{*}}{\left(g^{a^{q+2-i}}\right)}^{\gamma_{i}}\\ & =g^{\alpha }{\left(g^{a}\right)}^{\hat{r}+\sum_{i=1}^{n^{*}}{\left(g^{a^{q+1-i}}\right)}^{\gamma_{i}}}=g^{\alpha }g^{ar_{d}}, \end{array}$$
  $$\begin{array}{cc} K_{d,x} & =L_{d}^{f_{x}}\cdot \prod_{j=1}^{n^{*}}{\left(g^{a^{j}\cdot \hat{r}}\prod_{\underset{o\neq j}{o=1,\ldots ,n^{*}}}{\left(g^{a^{q+1+j-o}}\right)}^{\gamma_{o}}\right)}^{M_{i,j}^{*}}=g^{\hat{r}f_{x}}\prod_{i=1}^{n^{*}}{\left(g^{a^{q+1-i}}\right)}^{\gamma_{i}f_{x}}\cdot \prod_{j=1}^{n^{*}}{\left(g^{a^{j}}\right)}^{\hat{r}\cdot M_{i,j}^{*}}\\ & ={\left(g^{f_{x}}\prod_{j=1}^{n^{*}}{\left(g^{a^{j}}\right)}^{M_{i,j}^{*}}\right)}^{\hat{r}}\cdot \prod_{i=1}^{n^{*}}{\left(g^{a^{q+1-i}}\right)}^{\gamma_{i}f_{x}}=h_{x}^{\hat{r}}\cdot \prod_{i=1}^{n^{*}}{\left({h_{x}}^{a^{q+1-i}}\right)}^{\gamma_{i}}=h_{x}^{r_{d}}. \end{array}$$
- **Signcrypt queries**: When A issues a query on $\left(m,W_{e},W_{s},A_{d},A_{s}\right)$ and the cuckoo filter, if signing attribute set $A_{s}\in W_{s}$, B runs the **sExtract queries** and gets the private key $SK_{A_{s}}$, then B executes the **Signcrypt** algorithm, generates ciphertext $CT=\{C,C^{\prime },C^{\prime \prime },{\left\{C_{i}\right\}}_{i\in [1,l]},S_{1},S_{2},CF\}$. Finally, B returns *CT* to A.
- **Unsigncrypt queries**: When A issues a query on the ciphertext *CT*, B checks whether $C^{\prime }=C^{\prime *}$. If $C^{\prime }=C^{\prime *}$, B aborts. (Since $C^{\prime }=g^{\sigma }$ is random, the probability is at most 1/p). Otherwise, B first checks the corresponding attributes of EHR user are in cuckoo filter or not and reconstructs the encryption access policy $W_{e}^{\prime *}=\left(M_{e}^{*},\rho_{e}^{\prime *}\right)$.
  - If $A_{d}\notin W_{e}^{\prime *}$, B generates the private key $SK_{A_{d}}$ through executing the **dExtract queries** and returns the results of the **Unsigncrypt** algorithm to A.
  - If $A_{d}\in W_{e}^{\prime *}$, B first checks the validity of ciphertext CT based on Equation (3). If it is not valid, then B outputs ⊥; Otherwise computes $Y^{\sigma }=e{\left(C^{\prime \prime }/{C^{\prime }}^{\varsigma },g_{1}\right)}^{{\left(\frac{\mu }{\mu^{*}}-1\right)}^{-1}}\cdot e\left(C^{\prime },g^{\alpha^{\prime }}\right)$. Finally, B returns the message $m=\frac{C}{Y^{\sigma }}$ to A.
  **Correctness**:
  $$\begin{array}{cc} e\;\;{\left(C^{\prime \prime }/{C^{\prime }}^{\varsigma },g_{1}\right)}^{{\left(\frac{\mu }{\mu^{*}}-1\right)}^{-1}}\cdot e\left(C^{\prime },g^{\alpha^{\prime }}\right) & =e\left({\left(\delta_{1}^{\mu }\delta_{2}\right)}^{\sigma }/g^{\sigma \varsigma },g_{1}\right)\;{\left(\frac{\mu }{\mu^{*}}-1\right)}^{-1}\cdot e\left(C^{\prime },g^{\alpha^{\prime }}\right)\\ & =\left({\left({\left({g_{q}}^{\frac{1}{\mu^{*}}}\right)}^{\mu^{*}}\cdot g^{\varsigma }g_{q}^{-1}\right)}^{\sigma }/g^{\sigma \varsigma },g_{1}\right){\left(\frac{\mu }{\mu^{*}}-1\right)}^{-1}\cdot e\left(C^{\prime },g^{\alpha^{\prime }}\right)\\ & =e\left(g_{q}^{\sigma },g_{1}\right)\cdot e\left(C^{\prime },g^{\alpha^{\prime }}\right)\\ & =e\left(C^{\prime },g^{a^{n+1}}\right)\cdot e\left(C^{\prime },g^{\alpha^{\prime }}\right)\\ & =e(C^{\prime },g^{\alpha }). \end{array}$$
  Since Equation (3) is valid, it has $e\left(g^{ar_{d}},C^{\prime }\right)=\prod_{i\in I}e{\left(g^{ar_{d}},g\right)}^{\lambda_{i}\omega_{i}}$. Therefore,
  $$\begin{array}{cc} & \;e\;\;{\left(C^{\prime \prime }/{C^{\prime }}^{\varsigma },g_{1}\right)}^{{\left(\frac{\mu }{\mu^{*}}-1\right)}^{-1}}\cdot e\left(C^{\prime },g^{\alpha^{\prime }}\right)\\ = & e\left(C^{\prime },g^{\alpha }\right)\cdot \frac{e(g^{ar_{d}},C^{\prime })}{\prod_{i\in I}e{\left(g^{ar_{d}},g\right)}^{\lambda_{i}\omega_{i}}}=\frac{e(C^{\prime },g^{\alpha }g^{ar_{d}})}{\prod_{i\in I}{\left(e\left(g^{a\lambda_{i}}h_{\rho_{e}\left(i\right)}^{-\sigma },g^{r_{d}}\right)\cdot e\left(g^{\sigma },h_{\rho_{e}\left(i\right)}^{r_{d}}\right)\right)}^{\omega_{i}}}\\ = & \frac{e(C^{\prime },K_{d})}{\prod_{i\in I}{\left(e\left(C_{i},L_{d}\right)\cdot e\left(C^{\prime },K_{d,x}\right)\right)}^{\omega_{i}}}=Y^{\sigma }. \end{array}$$

**Challenge**: A outputs two equal length messages $m_{0}^{*},m_{1}^{*}\in M$ and the signing access policy $W_{s}^{*}$ to B. B chooses $t_{1}^{\prime }=0$, $\tilde{r},t_{2}^{\prime },t_{3}^{\prime },\cdots ,t_{n^{*}}^{\prime }\in Z_{p}^{*}$ and sets $r_{s}=\tilde{r}-a^{q}$, $\vec{v}=\left(\sigma +t_{1}^{\prime },\sigma a+t_{2}^{\prime },\sigma a^{2}+t_{3}^{\prime },\cdots ,\sigma a^{n^{*}-1}+t_{n^{*}}^{\prime }\right)=\sigma \left(1,a,a^{2},\cdots ,a^{n^{*}-1}\right)+\left(0,t_{2}^{\prime },t_{3}^{\prime },\cdots ,t_{n^{*}}^{\prime }\right)$. Then B selects θ∈{0,1} and outputs the challenge ciphertext $CT^{*}=\left(C^{*},C^{\prime *},C^{\prime \prime *},{\left\{{C_{i}}^{*}\right\}}_{i\in [1,l^{*}]},S_{1}^{*},S_{2}^{*}\right)$ as follows:

- $C^{*}=m_{\theta }T\cdot e\left(g^{\sigma },g^{a^{\prime }}\right)$,
- $C^{\prime *}=g^{\sigma }$,
- $C^{\prime \prime *}={\left(g^{\sigma }\right)}^{\varsigma }$, where $\mu^{*}=H_{2}\left(C^{\prime *}\right)$,
- ${C_{i}}^{*}=\left(\prod_{j=1}^{n^{*}}{\left(g^{a}\right)}^{M_{i,j}^{*}\cdot {t^{\prime }}_{j}}\right)\cdot {\left(g^{\sigma }\right)}^{-f_{\rho_{e}^{*}\left(i\right)}}$ for $i\in [1,l^{*}]$,
- $S_{1}^{*}=g^{\tilde{r}}g_{q}^{-1}$,
- $S_{2}^{*}=\left(g^{\alpha^{\prime }}g^{a\tilde{r}}\right)\cdot {\left(h_{\rho_{s}^{*}\left(i\right)}^{r^{*}}g_{q}^{-f_{\rho_{s}^{*}\left(i\right)}}\right)}^{\varphi_{i}^{*}}\cdot {\left(g^{s}\right)}^{\eta_{0}+\sum_{i=1}^{l^{*}}j_{i}^{*}\eta_{i}+\varsigma \xi \beta^{*}}$, where $H_{1}\left(S_{1}^{*},W_{e}^{*},W_{s}^{*}\right)=\left(j_{1}^{*},j_{2}^{*},\cdots ,{j_{l}}^{*}\right)$, $H_{3}\left(W_{e}^{*},W_{s}^{*},C,C^{\prime *},C^{\prime \prime *},{\left\{{C_{i}}^{*}\right\}}_{i\in [1,l^{*}]}\right)=\beta^{*}$.

If $T=e(g^{\sigma },g^{a^{q+1}})$, $CT^{*}$ is a valid challenge ciphertext.

**Correctness**:

$$T\cdot e\left(g^{\sigma },g^{\alpha^{\prime }}\right)=e\left(g^{\sigma },g^{a^{q+1}}\right)\cdot e\left(g^{\sigma },g^{\alpha^{\prime }}\right)=e{\left(g,g\right)}^{\sigma \alpha }=Y^{\sigma }.$$

$$C^{*}=m_{\theta }\cdot T\cdot e\left(g^{\sigma },g^{a^{\prime }}\right)=m_{\theta }\cdot Y^{\sigma }.$$

$$C^{\prime \prime *}={\left(g^{\sigma }\right)}^{\varsigma }={\left(g^{\varsigma }g_{q}^{-1}g_{q}\right)}^{\sigma }={\left({\left({g_{q}}^{\frac{1}{\mu^{*}}}\right)}^{\mu^{*}}\cdot g^{\varsigma }g_{q}^{-1}\right)}^{\sigma }={\left(\delta_{1}^{\mu^{*}}\delta_{2}\right)}^{\sigma }.$$

For $j=1,2,\cdots ,n^{*}$, $\lambda_{i}=\vec{v}\cdot {M_{i}}^{*}=\left(\sigma \left(1,a,a^{2},\cdots ,a^{n^{*}-1}\right)+\left(0,t_{2}^{\prime },t_{3}^{\prime },\cdots ,t_{n^{*}}^{\prime }\right)\right)\cdot {M_{i}}^{*}=a\sigma \sum_{j=1}^{n^{*}}a^{j-1}M_{i,j}^{*}+\sum_{j=2}^{n^{*}}{t^{\prime }}_{j}M_{i,j}^{*}$,

$$\begin{array}{cc} {C_{i}}^{*} & =\left(\prod_{j=1}^{n^{*}}{\left(g^{a}\right)}^{M_{i,j}^{*}\cdot {t^{\prime }}_{j}}\right)\cdot {\left(g^{\sigma }\right)}^{-f_{\rho_{e}^{*}\left(i\right)}}\\ & =g^{\sigma \sum_{j=1}^{n^{*}}a^{j}M_{i,j}^{*}}\cdot \left(\prod_{j=1}^{n^{*}}{\left(g^{a}\right)}^{M_{i,j}^{*}\cdot {t^{\prime }}_{j}}\right)\cdot {\left(g^{\sigma }\right)}^{-f_{\rho_{e}^{*}\left(i\right)}}\cdot g^{-\sigma \sum_{j=1}^{n^{*}}a^{j}M_{i,j}^{*}}\\ & =\left({\left(g^{a}\right)}^{\sigma \sum_{j=1}^{n^{*}}a^{j-1}M_{i,j}^{*}}\right)\cdot \left(\prod_{j=1}^{n^{*}}{\left(g^{a}\right)}^{M_{i,j}^{*}\cdot {t^{\prime }}_{j}}\right)\cdot {\left(g^{f_{\rho_{e}^{*}\left(i\right)}}\right)}^{-\sigma }\cdot g^{-\sigma \sum_{j=1}^{n^{*}}a^{j}M_{i,j}^{*}}\\ & =g^{a\lambda_{i}}\cdot {\left(g^{f_{\rho_{e}^{*}\left(i\right)}}g^{\sum_{j=1}^{n^{*}}a^{j}M_{i,j}^{*}}\right)}^{-\sigma }=g^{a\lambda_{i}}h_{\rho_{e}^{*}\left(i\right)}^{-\sigma }. \end{array}$$

$S_{1}^{*}=g^{\tilde{r}}g_{q}^{-1}=g^{\tilde{r}}g^{-a^{q}}=g^{r_{s}}=L_{s}$.

$$\begin{array}{cc} S_{2}^{*} & =\left(g^{\alpha^{\prime }}g^{a\tilde{r}}\right)\cdot {\left(h_{\rho_{s}^{*}\left(i\right)}^{r^{*}}g_{q}^{-f_{\rho_{s}^{*}\left(i\right)}}\right)}^{\varphi_{i}^{*}}\cdot {\left(g^{s}\right)}^{\eta_{0}+\sum_{i=1}^{l^{*}}j_{i}^{*}\eta_{i}+\varsigma \xi \beta^{*}}\\ & =g^{\alpha^{\prime }}g^{a^{q+1}}g^{a\tilde{r}}g^{-a^{q+1}}{\left(h_{\rho_{s}^{*}\left(i\right)}^{\tilde{r}}h_{\rho_{s}^{*}\left(i\right)}^{-a^{q}}\right)}^{\varphi_{i}^{*}}\cdot {\left(g^{\eta_{0}+\sum_{i=1}^{l^{*}}j_{i}^{*}\eta_{i}}\right)}^{\sigma }\cdot {\left({\left(g^{\sigma }\right)}^{\varsigma }\right)}^{\beta^{*}\xi }\\ & =g^{\alpha }g^{ar_{s}}\cdot h_{\rho_{s}^{*}\left(i\right)}^{\varphi_{i}^{*}}\cdot {\left(y_{0}\prod_{i=1}^{l^{*}}y_{i}^{j_{i}^{*}}\right)}^{\sigma }\cdot {\left({C^{\prime \prime }}^{*}\right)}^{\beta^{*}\xi }\\ & =K_{s}\cdot {\left(K_{s,x}\right)}^{\varphi_{i}^{*}}\cdot {\left(y_{0}\prod_{i=1}^{l^{*}}y_{i}^{j_{i}^{*}}\right)}^{\sigma }\cdot {\left({C^{\prime \prime }}^{*}\right)}^{\beta^{*}\xi }. \end{array}$$

**Phase 2**: A performs a series of queries as **Phase 1** except the **dExtract queries** on any decryption attribute set $A_{d}\in W_{e}^{*}$ and the **Unsigncrypt queries** on the challenge ciphertext $CT^{*}$ for any $A_{d}\in W_{e}^{*}$.

**Guess**: A outputs a guess bit $\theta^{\prime }\in \left\{0,1\right\}$. If $\theta^{\prime }=\theta$, B outputs 1 ($T=e{\left(g,g\right)}^{a^{q+1}\sigma }$); Otherwise B outputs 0 (T=R).

B can’t successfully simulate with aborting the game when the ciphertext satisfies $C^{\prime }=C^{\prime *}$ in the **Unsigncrypt queries**, the probability of this aborting event is at most $\frac{q_{us}}{p}$. If B doesn’t abort and $T=e{\left(g,g\right)}^{a^{q+1}\sigma }$, the probability of the successful simulation for B is at least $\frac{1}{2}+\varepsilon -\frac{q_{us}}{p}$. If T=R, the probability of A does not get any information about $m_{\theta }^{*}$ is $\frac{1}{2}$. Therefore, the advantage of B can solve the *q*-DBDHE problem is at least $\varepsilon^{\prime }=\mathrm{Pr}|B\left(y,T=e{\left(g,g\right)}^{a^{q+1}\sigma }\right)=0|\;\;-\mathrm{Pr}\;|B\left(y,T=R\right)=0|=\varepsilon -\frac{q_{us}}{p}$.

### 6.2. Unforgeability

**Theorem** **2.** *Assuming there is the adversary A who is capable of breaking the EUF-CMA security of CP-ABSC scheme with the non-negligible probability ε, then we can construct an algorithm B that can solve *q*-CDHE problem with the probability $\varepsilon^{\prime }=\varepsilon k\left(l+1\right)$, where k is the security parameter and l is the outputs length of hash function $H_{1}$.*

**Proof.**  B receives an instance $y_{a}=\left(g,g^{a},g^{a^{2}},\cdots ,g^{a^{q}},g^{a^{q+2}},\cdots ,g^{a^{2q}}\right)$ of the *q*-CDHE problem, where $a\in Z_{p}$, *g* is a generator of G and $g_{i}=g^{a^{i}}$. The goal of the algorithm B is to calculate $g^{a^{q+1}}$. B chooses three collision-resistant hash functions $H_{1}:{\left\{0,1\right\}}^{*}\to {\left\{0,1\right\}}^{l}$, $H_{2}:G\to Z_{p}^{*}$, $H_{3}:\left\{0,1\right\}\to Z_{p}^{*}$ and a one-way hash function $H_{4}:\left\{0,1\right\}\to Z_{p}^{*}$. B simulates the challenger in EUF-CMA security game and interacts with A as below. □

**Initialization**: A submits the challenge signing access policy $W_{s}^{*}=\left(M_{s}^{*},\rho_{s}^{*}\right)$ to B, where $M_{s}^{*}$ is a matrix of $l^{*}\times n^{*}$ with the labeling function $\rho_{s}^{*}$. Let ${\vec{M}}_{i}^{*}=\left(M_{i,1}^{*},M_{i,2}^{*},\cdots ,M_{i,n^{*}}^{*}\right)$ be the *i*’th row of $M_{s}^{*}$.

**Setup**: B randomly picks $\alpha^{\prime }\in Z_{p}^{*}$, $d,d^{\prime }\in Z_{p}^{*}$ and defines $\alpha =\alpha^{\prime }+a^{q+1}$, $Y=e\;\;{\left(g,g\right)}^{\alpha }=e\left(g^{a},g^{a^{q}}\right)\cdot e\;\;{\left(g,g\right)}^{\alpha^{\prime }}$, $\delta_{1}=g^{d}$, $\delta_{2}=g^{d^{\prime }}$. B randomly chooses $\left(z_{0},z_{1},\cdots ,z_{l}\right)\in Z_{p}^{l+1}$, η=k and η(l+1)<p, where *k* is a security parameter. B also randomly selects 0≤π≤l and $\left(b_{0},b_{1},\cdots ,b_{l}\right)\in Z_{\eta }^{l+1}$ sets $y_{0}=g_{q}^{p-\eta \pi +b_{0}}$, $y_{i}=g_{q}^{b_{i}}g^{z_{i}}$ for all i∈[1,l]. For each vector $\vec{j}=\left(j_{1},j_{2},\cdots ,j_{l}\right)\in {\left\{0,1\right\}}^{l}$, B defines two functions $F_{1}\left(\vec{j}\right)=p-\eta \pi +b_{0}+\sum_{i=1}^{l}j_{i}b_{i}$ and $F_{2}\left(\vec{j}\right)=z_{0}+\sum_{i=1}^{l}j_{i}z_{i}$, which means that $y_{0}\prod_{i=1}^{l}{y_{i}}^{j_{i}}=g_{q}^{F_{1}\left(\vec{j}\right)}g^{F_{2}\left(\vec{j}\right)}$. B defines the function $F:{\left\{0,1\right\}}^{l}\to \left\{0,1\right\}$ by $F\left(\vec{j}\right)=\left\{\begin{array}{cc} 0, & \mathrm{if}\;b_{0}+\sum_{i=1}^{l}j_{i}b_{i}=0\;\mathrm{mod}\;\eta ,\\ 1, & \mathrm{otherwise}. \end{array}\right.$. It can be seen that, if $F(\vec{j})=1$, then $F_{1}\left(\vec{j}\right)\neq 0\;\mathrm{mod}\;p$.

Finally, for each attribute x∈S, let *X* denote the set of indices *i*, such that $\rho_{s}^{*}\left(i\right)=x$. If X≠∅, B selects a random $f_{x}\in Z_{p}^{*}$ and defines $h_{x}=g^{f_{x}}\cdot g^{aM_{i,1}^{*}}\cdot g^{a^{2}M_{i,2}^{*}}\cdots g^{a^{n^{*}}M_{i,n}^{*}}$. If X=∅, then $h_{x}=g^{f_{x}}$.

B returns the public parameters $PK=\{S,M,H_{1},H_{2},H_{3},H_{4},Y,\delta_{1},\delta_{2},y_{0},{\left\{y_{i}\right\}}_{i\in [1,l]},Y,{\left\{h_{x}\right\}}_{x\in S}\}$ to A.

**Query phase**: A adaptively performs a number of polynomial bounded queries as follows.

- **sExtract queries**: When A issues a query on the signing attribute set $A_{s}$, if $A_{s}\notin W_{s}^{*}$, B randomly selects $\hat{r}\in Z_{p}^{*}$ and calculates the vector $\vec{\gamma }=\left(\gamma_{1},\gamma_{2},\cdots ,\gamma_{n^{*}}\right)\in Z_{p}^{n^{*}}$ where $\gamma_{1}=-1$ such that $\vec{\gamma }\cdot M_{i}^{*}=0$ for all *i* where $\rho_{s}^{*}\left(i\right)\in A_{s}$. B implicitly defines $r_{s}=\hat{r}+\gamma_{1}a^{q}+\gamma_{2}a^{q-1}+\cdots +\gamma_{n^{*}}a^{q-n^{*}+1}$ and computes $L_{s}=g^{\hat{r}}\prod_{i=1}^{n^{*}}{\left(g^{a^{q+1-i}}\right)}^{\gamma_{i}}$, $K_{s}=g^{\alpha^{\prime }}g^{a\hat{r}}\prod_{i=2}^{n^{*}}{\left(g^{a^{q+2-i}}\right)}^{\gamma_{i}}$ and $K_{s,x}=L_{s}^{f_{x}}\prod_{j=1}^{n^{*}}{\left(g^{a^{j}\cdot \hat{r}}\prod_{\underset{o\neq j}{o=1,\cdots ,n^{*}}}{\left(g^{a^{q+1+j-o}}\right)}^{\gamma_{o}}\right)}^{M_{i,j}^{*}}$ for any $x\in A_{s}$. If $\rho_{s}^{*}\left(i\right)\neq x$ for all *i*, B simply sets $K_{s,x}=L_{s}^{f_{x}}$. Then B returns the signing key $SK_{A_{s}}=\left\{L_{s},K_{s},{\left\{K_{s,x}\right\}}_{x\in A_{s}}\right\}$ to A.
  **Correctness**:
  $$L_{s}=g^{\hat{r}}\prod_{i=1}^{n^{*}}{\left(g^{a^{q+1-i}}\right)}^{\gamma_{i}}=g^{r_{s}},$$
  $$\begin{array}{cc} K_{s} & =g^{\alpha^{\prime }}g^{a\hat{r}}\prod_{i=2}^{n^{*}}{\left(g^{a^{q+2-i}}\right)}^{\gamma_{i}}=g^{\alpha^{\prime }}g^{a^{q+1}}\cdot g^{a\hat{r}}\cdot g^{-a^{q+1}}\prod_{i=2}^{n^{*}}{\left(g^{a^{q+2-i}}\right)}^{\gamma_{i}}=g^{\alpha }g^{a\hat{r}}\prod_{i=1}^{n^{*}}{\left(g^{a^{q+2-i}}\right)}^{\gamma_{i}}\\ & =g^{\alpha }{\left(g^{a}\right)}^{\hat{r}+\sum_{i=1}^{n^{*}}{\left(g^{a^{q+1-i}}\right)}^{\gamma_{i}}}=g^{\alpha }g^{ar_{s}}, \end{array}$$
  $$\begin{array}{cc} K_{s,x} & ={L_{s}}^{f_{x}}\cdot \prod_{j=1}^{n^{*}}{\left(g^{a^{j}\cdot \hat{r}}\prod_{\underset{k\neq j}{o=1,\ldots ,n^{*}}}{\left(g^{a^{q+1+j-o}}\right)}^{\gamma_{o}}\right)}^{M_{i,j}^{*}}=g^{\hat{r}f_{x}}\prod_{i=1}^{n^{*}}{\left(g^{a^{q+1-i}}\right)}^{\gamma_{i}f_{x}}\cdot \prod_{j=1}^{n^{*}}{\left(g^{a^{j}}\right)}^{\hat{r}\cdot M_{i,j}^{*}}\\ & ={\left(g^{f_{x}}\prod_{j=1}^{n^{*}}{\left(g^{a^{j}}\right)}^{M_{i,j}^{*}}\right)}^{\hat{r}}\cdot \prod_{i=1}^{n^{*}}{\left(g^{a^{q+1-i}}\right)}^{\gamma_{i}f_{x}}=h_{x}^{\hat{r}}\cdot \prod_{i=1}^{n^{*}}{\left({h_{x}}^{a^{q+1-i}}\right)}^{\gamma_{i}}=h_{x}^{r_{s}}. \end{array}$$
- **dExtract queries**: When A issues a query on the decryption attribute set $A_{d}$, B randomly picks $\hat{r}\in Z_{p}^{*}$, sets $r_{d}=\hat{r}-a^{q}$ and computes $L_{d}=g^{\hat{r}}g_{q}^{-1}$, $K_{d}=g^{\alpha^{\prime }}g_{1}^{\hat{r}}$ and $K_{d,x}=h_{x}^{\hat{r}}g_{q}^{-f_{x}}$ for any $x\in A_{d}$. Then B returns the decryption private key $SK_{A_{d}}=\left\{L_{d},K_{d},{\left\{K_{d,x}\right\}}_{x\in A_{d}}\right\}$ to A.
  **Correctness**:
  $$L_{d}=g^{\hat{r}}g_{q}^{-1}=g^{\hat{r}}g^{-a^{q}}=g^{r_{d}},$$
  $$K_{d}=g^{\alpha^{\prime }}g_{1}^{\hat{r}}=g^{\alpha^{\prime }}g_{q+1}g_{1}^{\hat{r}}g_{q+1}^{-1}=g^{\alpha^{\prime }+a^{q+1}}g^{a\hat{r}-a^{q+1}}=g^{\alpha }g^{ar_{d}},$$
  $$K_{d,x}=h_{x}^{\hat{r}}g_{q}^{-f_{x}}=h_{x}^{\hat{r}}{\left(h_{x}\right)}^{-a^{q}}=h_{x}^{r_{d}}.$$
- **Signcrypt queries**: When A issues a query on $\left(m,W_{e},W_{s},A_{d},A_{s}\right)$ and the cuckoo filter,
  - If $A_{s}\notin W_{s}^{*}$, B gets the private key $SK_{A_{s}}$ by running the **sExtract queries**. Then B generates ciphertext *CT* by executing the **Signcrypt** algorithm and returns to A.
  - If $A_{s}\in W_{s}^{*}$, B performs the following steps: B randomly chooses $\varphi_{i}\in Z_{p}^{l}$ and generates a vector $\vec{\varphi }=\left(-\varphi_{1},-\varphi_{2},\cdots ,-\varphi_{l}\right)$ such that $\vec{\varphi }\cdot M_{s}=-{\vec{1}}_{n}$, that is $\sum_{i=1}^{l}\varphi_{i}\cdot M_{s,i}=-{\vec{1}}_{n}$, and $\varphi_{i}=0$ for all i∈[1,l], where $\rho_{s}\left(i\right)\notin A_{s}$. B sets $C=mY^{\sigma }$, $S_{1}=g^{r_{s}}$ and computes $\vec{j}=\left(j_{1},j_{2},\cdots ,j_{l}\right)=H_{1}\left(S_{1},W_{e},W_{s}\right)$. If $F(\vec{j})=0$, B aborts; Otherwise, B chooses a random number $\sigma^{\prime }\in Z_{p}^{*}$, sets $\sigma =\sigma^{\prime }-\frac{a}{F_{1}\left(\vec{j}\right)}$ and computes $C^{\prime }=g^{\sigma^{\prime }}{g_{1}}^{-1/F_{1}\left(\vec{j}\right)}$, $C^{\prime \prime }=g^{\left(d\mu +d^{\prime }\right)\sigma^{\prime }}{g_{1}}^{-(\mu d+d^{\prime })/F_{1}\left(\vec{j}\right)}$, where $\mu =H_{2}\left(C^{\prime }\right)$. B randomly chooses $v_{2},\cdots ,v_{n}\in Z_{p}^{*}$ and defines $\vec{v}=\left(\sigma^{\prime }-\frac{a}{F_{1}\left(\vec{j}\right)},v_{2},\cdots ,v_{n}\right)$ and $\lambda_{i}=\vec{v}\cdot M_{i}=\left(\sigma^{\prime }-\frac{a}{F_{1}\left(\vec{j}\right)}\right)M_{i,1}+\sum_{i=2}^{l}v_{i}M_{i,n}$ for all i∈[1,n]. B sets $C_{i}={g_{1}}^{\left(\sigma^{\prime }M_{i,1}+\sum_{i=2}^{l}v_{i}M_{i,n}\right)}\cdot {g_{2}}^{-M_{i,1}/F_{1}\left(\vec{j}\right)}\cdot {h_{\rho (i)}}^{-\sigma^{\prime }}\cdot {g_{1}}^{f_{\rho_{e}\left(i\right)}/F_{1}\left(\vec{j}\right)}$ for i∈[1,l], $S_{2}=g^{\alpha^{\prime }}g^{ar_{s}}\left(\prod_{i=1}^{l}{\left(h_{\rho_{s}\left(i\right)}^{r_{s}}\right)}^{\varphi_{i}}\right)\cdot {\left({g_{q}}^{F_{1}\left(\vec{j}\right)}g^{F_{2}\left(\vec{j}\right)}\right)}^{\sigma^{\prime }}\cdot \left({g_{1}}^{-F_{2}\left(\vec{j}\right)/F_{1}\left(\vec{j}\right)}\right)\cdot {\left(C^{\prime \prime }\right)}^{\beta \xi }$, where $\beta =H_{3}\left(W_{e},W_{s},C,C^{\prime },C^{\prime \prime },{\left\{C_{i}\right\}}_{i\in [1,l]}\right)$. Finally, B returns the ciphertext $CT=\{C,C^{\prime },C^{\prime \prime },{\left\{C_{i}\right\}}_{i\in [1,l]},S_{1},S_{2},CF\}$ to A.
  **Correctness**:
  $$C^{\prime }=g^{\sigma^{\prime }}{g_{1}}^{-1/F_{1}\left(\vec{j}\right)}=g^{\sigma^{\prime }-a/F_{1}\left(\vec{j}\right)}=g^{\sigma },$$
  $$\begin{array}{cc} C_{i} & ={g_{1}}^{\left(\sigma^{\prime }M_{i,1}+\sum_{i=2}^{l}v_{i}M_{i,n}\right)}\cdot {g_{2}}^{-M_{i,1}/F_{1}\left(\vec{j}\right)}\cdot {h_{\rho_{e}\left(i\right)}}^{-\sigma^{\prime }}\cdot {g_{1}}^{f_{\rho_{e}\left(i\right)}/F_{1}\left(\vec{j}\right)}\\ & =g^{a^{\left(\sigma^{\prime }M_{i,1}+\sum_{i=2}^{l}v_{i}M_{i,n}\right)}}\cdot {\left(g^{a}\right)}^{-aM_{i,1}/F_{1}\left(\vec{j}\right)}\cdot {h_{\rho_{e}\left(i\right)}}^{-\left(\sigma^{\prime }-a/F_{1}\left(\vec{j}\right)\right)}=g^{a\lambda_{i}}h_{\rho_{e}\left(i\right)}^{-\sigma }, \end{array}$$
  $$\begin{array}{cc} S_{2} & =g^{\alpha^{\prime }}g^{ar_{s}}\left(\prod_{i=1}^{l}{\left(h_{\rho_{s}\left(i\right)}^{r_{s}}\right)}^{\varphi_{i}}\right)\cdot {\left({g_{q}}^{F_{1}\left(\vec{j}\right)}g^{F_{2}\left(\vec{j}\right)}\right)}^{\sigma^{\prime }}\cdot \left({g_{1}}^{-F_{2}\left(\vec{j}\right)/F_{1}\left(\vec{j}\right)}\right)\cdot {\left(C^{\prime \prime }\right)}^{\beta \xi }\\ & =g^{\alpha^{\prime }}g^{a^{q+1}}g^{ar_{s}}\cdot {\left({g_{q}}^{F_{1}\left(\vec{j}\right)}g^{F_{2}\left(\vec{j}\right)}\right)}^{\sigma^{\prime }}\cdot g^{-a^{q+1}}\cdot \left({g_{1}}^{-F_{2}\left(\vec{j}\right)/F_{1}\left(\vec{j}\right)}\right)\cdot {\left(C^{\prime \prime }\right)}^{\beta \xi }\\ & =\left(g^{\alpha }g^{ar_{s}}\right)\cdot \left(\prod_{i=1}^{l}{\left(h_{\rho_{s}\left(i\right)}^{r_{s}}\right)}^{\varphi_{i}}\right)\cdot {\left({g_{q}}^{F_{1}\left(\vec{j}\right)}g^{F_{2}\left(\vec{j}\right)}\right)}^{\sigma^{\prime }}\cdot {\left({g_{q}}^{F_{1}\left(\vec{j}\right)}g^{F_{2}\left(\vec{j}\right)}\right)}^{-a/F_{1}\left(\vec{j}\right)}\cdot {\left(C^{\prime \prime }\right)}^{\beta \xi }\\ & =K_{s}\cdot {\left(K_{s,x}\right)}^{\varphi_{i}}\cdot (y_{0}\prod_{i=1}^{l}{y_{i}}^{j_{i}}{)}^{\sigma }\cdot {\left(C^{\prime \prime }\right)}^{\beta \xi }. \end{array}$$
- **Unsigncrypt queries**: When A issues a query on the ciphertext *CT*, B computes the decryption private key $SK_{A_{d}}$ by executing the **dExtract queries**. Then B generates the message *m* by executing the **Unsigncrypt** algorithm and returns to A.

**Forgery**: A outputs the valid forgery ciphertext $CT^{*}=\left\{C^{*},C^{\prime *},C^{\prime \prime *},{\left\{C_{i}^{*}\right\}}_{i\in [1,l]},S_{1}^{*},S_{2}^{*},CF\right\}$ on $\left(m^{*},W_{e}^{*},W_{s}^{*}\right)$. $CT^{*}$ satisfies the following two conditions:

1. Since $A_{d}^{*}\in W_{e}^{*}$, the result of the **Unsigncrypt** algorithm is $m^{*}\neq ⊥$;
2. A never issues the **Signcrypt queries** on $\left(m^{*},W_{e}^{*},W_{s}^{*}\right)$.

Now, B could provide the methods to solve the *q*-CDHE problem as follows.

Firstly, B computes ${\vec{j}}^{*}=\left(j_{1}^{*},j_{2}^{*},\cdots ,j_{l}^{*}\right)=H_{1}\left(S_{1}^{*},W_{e}^{*},W_{s}^{*}\right)$. If $b_{0}+\sum_{i=1}^{l}j_{i}b_{i}\neq \eta \pi$, then B aborts. Otherwise, $F_{1}\left({\vec{j}}^{*}\right)=0\;\;\;\;\mathrm{mod}\;\;\;\;\;p$, B computes $C^{*}=m^{*}Y^{\sigma }$, $C^{\prime *}=g^{\sigma }$, $C^{\prime \prime *}=g^{(d\mu +d^{\prime })\sigma }$, ${\left\{C_{i}^{*}=g^{a\lambda_{i}}h_{\rho_{e}^{*}\left(i\right)}^{\sigma }\right\}}_{i\in [1,l^{*}]}$, $S_{1}^{*}=g^{r_{s}}$, $S_{2}^{*}=g^{\alpha }g^{ar_{s}}\left(\prod_{i=1}^{l^{*}}{\left(h_{\rho_{s}^{*}\left(i\right)}^{r_{s}}\right)}^{\varphi_{i}^{*}}\right){\left(y_{0}\prod_{i\in [1.l]}{y_{i}}^{j_{i}^{*}}\right)}^{\sigma }\cdot {\left(g^{\left(d\mu +d^{\prime }\right)}\right)}^{\sigma \xi \beta^{*}}$, where $\mu^{*}=H_{2}\left(C^{\prime *}\right)$, $\beta^{*}=H_{3}\left(W_{e}^{*},W_{s}^{*},C^{*},C^{\prime *},C^{\prime \prime *},{\left\{C_{i}^{*}=g^{a\lambda_{i}}h_{\rho_{e}^{*}\left(i\right)}^{\sigma }\right\}}_{i\in [1,l^{*}]}\right)$ and the vector ${\vec{\varphi }}^{*}=\left(-\varphi_{1},-\varphi_{2},\cdots ,-\varphi_{l^{*}}\right)$ satisfies $\sum_{i=1}^{l^{*}}\varphi^{*}\cdot M_{s,i}^{*}=-{\vec{1}}_{n^{*}}$.

Then B can calculate $\frac{S_{2}^{*}}{g^{\alpha }\left(\prod_{i=1}^{l^{*}}{\left(S_{1}^{*}\right)}^{f_{\rho_{s}^{*}\left(i\right)}}\right){\left({C^{\prime }}^{*}\right)}^{F_{2}\left({\vec{j}}^{*}\right)+\left(d\mu +d^{\prime }\right)\xi \beta^{*}}}=g^{a^{q+1}}$.

**Correctness**:$\sum_{i=1}^{l^{*}}\varphi_{i}^{*}\cdot M_{s,i}^{*}=-{\vec{1}}_{n^{*}}$ implies $\sum_{i=1}^{l^{*}}\varphi_{i}^{*}\cdot M_{i,j}^{*}=\left\{\begin{array}{c} -1,j=1;\\ 0,if2\leq j\leq n^{*} \end{array}\right.$, so $\sum_{i=1}^{l^{*}}\sum_{j=1}^{l_{*}}a^{j}M_{i,j}^{*}\varphi_{i}^{*}r_{s}=-ar_{s}$.

$$\begin{array}{c} \;\frac{S_{2}^{*}}{g^{\alpha }\left(\prod_{i=1}^{l^{*}}{\left(S_{1}^{*}\right)}^{f_{\rho_{s}^{*}\left(i\right)}}\right){\left({C^{\prime }}^{*}\right)}^{F_{2}\left({\vec{j}}^{*}\right)+\left(d\mu +d^{\prime }\right)\xi \beta^{*}}}\\ =\frac{g^{\alpha }g^{ar_{s}}\cdot \left(\prod_{i=1}^{l^{*}}h_{\rho_{s}^{*}\left(i\right)}^{r_{s}\varphi_{i}^{*}}\right){\left(y_{0}\prod_{i\in [1,l^{*}]}{y_{i}}^{j_{i}^{*}}\right)}^{\sigma }\cdot {\left(g^{\left(d\mu^{*}+d^{\prime }\right)}\right)}^{\sigma \xi \beta^{*}}}{g^{\alpha }\cdot \left(\prod_{i=1}^{l^{*}}{\left(S_{1}^{*}\right)}^{\varphi_{i}^{*}f_{\rho_{s}^{*}\left(i\right)}}\right)\cdot {\left({C^{\prime }}^{*}\right)}^{F_{2}\left({\vec{j}}^{*}\right)+\left(d\mu +d^{\prime }\right)\xi \beta^{*}}}\\ =\frac{g^{\alpha^{\prime }+a^{q+1}}g^{ar_{s}}\cdot \left(\prod_{i=1}^{l^{*}}{\left(g^{f_{\rho_{s}^{*}\left(i\right)}}\prod_{j=1}^{n}g^{a^{j}M_{i,j}^{*}}\right)}^{\varphi_{i}^{*}r_{s}}\right)\cdot {\left({g_{q}}^{F_{1}\left({\vec{j}}^{*}\right)}g^{F_{2}\left({\vec{j}}^{*}\right)}\right)}^{\sigma }\cdot {\left(g^{\sigma }\right)}^{\left(d+u^{*}d^{\prime }\right)\xi \beta^{*}}}{g^{\alpha }\cdot \left(\prod_{i=1}^{l^{*}}{\left(S_{1}^{*}\right)}^{\varphi_{i}^{*}f_{\rho_{s}^{*}\left(i\right)}}\right)\cdot {\left({C^{\prime }}^{*}\right)}^{F_{2}\left({\vec{j}}^{*}\right)+\left(d\mu +d^{\prime }\right)\xi \beta^{*}}}\\ =\frac{g^{\alpha^{\prime }}g^{a^{q+1}}g^{ar_{s}}\cdot \left(\prod_{i=1}^{l^{*}}{\left(g^{r_{s}}\right)}^{\varphi_{i}^{*}f_{\rho_{s}^{*}\left(i\right)}}\right)\cdot \left(\prod_{i=1}^{l^{*}}\prod_{j=1}^{n}g^{a^{j}M_{i,j}^{*}\varphi_{i}^{*}r_{s}}\right)\cdot {\left(g^{\sigma }\right)}^{F_{2}\left({\vec{j}}^{*}\right)+\left(d\mu +d^{\prime }\right)\xi \beta^{*}}}{g^{\alpha }\cdot \left(\prod_{i=1}^{l^{*}}{\left(S_{1}^{*}\right)}^{\varphi_{i}^{*}f_{\rho_{s}^{*}\left(i\right)}}\right)\cdot {\left({C^{\prime }}^{*}\right)}^{F_{2}\left({\vec{j}}^{*}\right)+\left(d\mu +d^{\prime }\right)\xi \beta^{*}}}\\ =\frac{g^{\alpha^{\prime }}g^{a^{q+1}}g^{ar_{s}}\cdot \left(\prod_{i=1}^{l^{*}}{\left(g^{r_{s}}\right)}^{\varphi_{i}^{*}f_{\rho_{s}^{*}\left(i\right)}}\right)\cdot g^{-ar_{s}}\cdot {\left({C^{\prime }}^{*}\right)}^{F_{2}\left({\vec{j}}^{*}\right)+\left(d\mu +d^{\prime }\right)\xi \beta^{*}}}{g^{\alpha }\left(\prod_{i=1}^{l^{*}}{\left(S_{1}^{*}\right)}^{\varphi_{i}^{*}f_{\rho_{s}^{*}\left(i\right)}}\right)\cdot {\left({C^{\prime }}^{*}\right)}^{F_{2}\left({\vec{j}}^{*}\right)+\left(d\mu +d^{\prime }\right)\xi \beta^{*}}}=g^{a^{q+1}}. \end{array}$$

In the Forgery phase, B can successfully simulate without aborting if $b_{0}+\sum_{j\in [1,l^{*}]}m_{j}^{*}b_{j}=\eta \pi$. The probability of this simulation is not abort is $\frac{1}{\eta }\frac{1}{l+1}=\frac{1}{k(l+1)}$. Therefore, the success probability of B for solving the *q*-CDHE problem is at least $\varepsilon^{\prime }=\varepsilon /k(l+1)$.

## 7. Performance Analysis

The functionality, computation and communication costs of the proposed CP-ABSC scheme are evaluated in this section. We also compare them with other related schemes [20,21,22,23].

### 7.1. Functionality Comparison

The functionality comparisons between the proposed CP-ABSC scheme and other related schemes [20,21,22,23] are presented. Let MC be the message confidentiality, CU be the ciphertext unforgeability, CPA be the chosen plaintext attacks, CCA be the chosen ciphertext attack, CMA be the chosen message attack, ROM be the random model and SM be the standard model. Table 1 summarizes the functionality comparison results.

It is clear from Table 1 that only the scheme [22] adopts the threshold policy as access policy which only supports simple predicates. Although the schemes [20,21] support monotone tree policy which can transform into LSSS access policy, the construction of this type of access structure is quite complicated. The scheme [23] and our proposed scheme support LSSS access structure that has the simpler construction process. In addition, our scheme and the schemes [21,23] can satisfy public verifiability. All schemes realize CCA security and CMA security in the standard model except [20]. In particular, none of these schemes [20,21,22,23] could provide the property of privacy-preserving, only our scheme protects the personal privacy of EHR owners.

### 7.2. Computation Cost

We analyze the computation cost of the proposed CP-ABSC scheme and compare it with that of other related schemes [20,21,22,23]. For computation complexity estimation, we define the following time cost for performing the cryptographic operations required in all schemes. Let $T_{p}$ be the time for performance a pairing, $T_{m}$ be the time for performance a scale multiplication in G, $T_{mt}$ be the time for performance a scale multiplication in $G_{T}$. Other lightweight operations (the arithmetic operation in $Z_{p}$, one-way hash function)are not taken into account.

To offer the security level to 80-bit, we adopt the symmetric bilinear pairing $e:G\times G\to G_{T}$, where G be the multiplicative cyclic group by *p*, *p* is 512-bit prime number. The simulation experiment is based on the C++ Pairing-Based Cryptography (PBC) library MIRACL and runs on Intel Core i5-4590, 3.3 GHz CPU, 8 gigabytes memory with Windows 7 environment.

In this paper, we execute the experiment on a common PC, if the experiment were to run in a practical cloud environment, such as EC2 cloud computing service [49], it would actually run faster. The average execution times of $T_{p}$, $T_{m}$ and $T_{mt}$ are listed in Table 2.

Let *l* be the number of attributes in attribute space. We summarize the computation costs of the proposed scheme, Wang et al.’s scheme [20], Emura et al.’s scheme [21], Hu et al.’s scheme [22] and Rao et al.’s scheme [23] in Table 3.

In terms of the **Signcrypt** phase, for the computation costs of *l* attributes, Wang et al.’s scheme [20] requires to execute (4l+3) scalar multiplication operations in G, two scalar multiplication operations in $G_{T}$ and one bilinear pairing operation. Therefore, the total signcryption time is 7.554l+23.5863 ms. Emura et al.’s scheme [21] needs to execute (6l+2) scalar multiplication operations in G and one scalar multiplication operation in $G_{T}$. Therefore, the total signcryption time is 15.108l+22.2587 ms. Hu et al.’s scheme [22] needs to execute (4l+2) scalar multiplication operations in G and one scalar multiplication operation in $G_{T}$. Therefore, the total signcryption time is 22.662l+23.5683 ms. Rao et al.’s scheme [23] needs to execute (5l+7) scalar multiplication operations in G and one scalar multiplication operation in $G_{T}$. Therefore, the total signcryption time is 15.108l+8.4783 ms. The proposed scheme needs to execute (2l+6) scalar multiplication operations in G and one scalar multiplication operation in $G_{T}$. Therefore, the total signcryption time is 18.885l+27.3633 ms.

In terms of the **Unsigncrypt** phase, for the computation costs of *l* attributes, Wang et al.’s scheme [20] needs to execute (2l+1) scalar multiplication operations in $G_{T}$ and (4l+4) bilinear pairing operations. Therefore, the total unsigncryption time is 38.165l+37.2407 ms. Emura et al.’s scheme [21] needs to execute (6l+3) bilinear pairing operations. Therefore, the total unsigncryption time is 54.4746l+27.2373 ms. Hu et al.’s scheme [22] needs to execute 2l scalar multiplication operations in $G_{T}$ and 5l bilinear pairing operations. Therefore, the total unsigncryption time is 47.2441l ms. Rao et al.’s scheme [23] needs to execute (3l+2) scalar multiplication operations in G and (l+5) bilinear pairing operations. Therefore, the total unsigncryption time is 20.4101l+52.9495 ms. The proposed scheme needs to execute four scalar multiplication operations in G, *l* scalar multiplication operations in $G_{T}$ and (2l+4) bilinear pairing operations. Therefore, the total unsigncryption time is 19.0825l+51.4244 ms.

**Figure 4:**
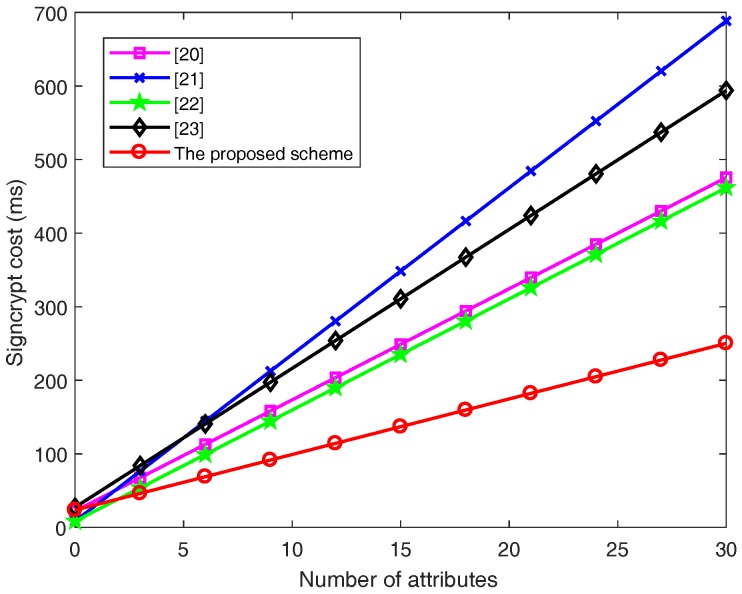
Signcrypt cost with the number of attributes.

**Figure 5:**
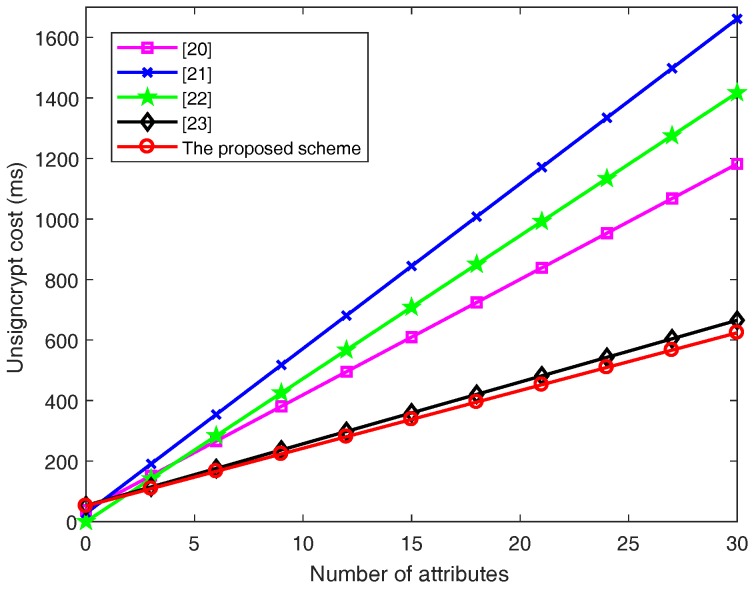
Unsigncrypt cost with the number of attributes.

Figure 4 and Figure 5 clearly illustrate the computation cost of the signcrypt and unsigncrypt phases with increasing number of attributes *l*, respectively.

From Figure 4 and Figure 5, the computation costs in both the signcrypt and unsigncrypt phases rise linearly with the number of attributes in all the schemes. It can be easily see that the proposed scheme’s slope is the lowest.

In Figure 4, for l=10, the computation cost of signcrypt is equal to 173.3387, 250.1883, 159.5583, 216.2133 and 99.1263 ms when the schemes [20,21,22,23] and the proposed scheme are adopted, respectively. For l=30, the computation cost of signcrypt is equal to 475.4987, 703.4283, 461.7183, 593.9133 and 250.2063 ms when the schemes [20,21,22,23] and the proposed scheme are adopted, respectively.

In Figure 5, for l=10, the computation costs of unsigncrypt is equal to 418.8907, 571.9833, 472.441, 257.0505 and 242.2494 ms when the schemes [20,21,22,23] and the proposed scheme are adopted, respectively. For l=30, the computation cost of unsigncrypt is equal to 1182.1907, 1661.4753, 1417.323, 665.2525 and 623.8994 ms when the schemes [20,21,22,23] and the proposed scheme are adopted, respectively.

According to Figure 4 and Figure 5, we intuitively obtain that the proposed scheme achieves the lowest computation cost with the increase of the number of attributes, especially after adding the cuckoo filter, without increasing extra computation costs in. Therefore, our proposed CP-ABSC scheme is efficient in both the signcrypt and unsigncrypt phase, which has much more advantages than the previous schemes [20,21,22,23].

### 7.3. Communication Cost

We discuss the communication cost of the proposed CP-ABSC scheme with other related schemes [20,21,22,23]. Let *l* be the number of attributes in attribute space, |G| be the element’s length in group G and $|G_{T}|$ be the element’s length in group $G_{T}$. Since the size of *p* is 512 bits (64 bytes), therefore the element’s size in group G and $G_{T}$ is 512 bits (64 bytes) and 3072 bits (384 bytes), respectively. We also take into account the communication costs of using cuckoo filter. Assume that we use the one-way hash function in cuckoo filter, and its outputs length is 160 bits (20 bytes). When the number of EHR owner’s attributes is *l*, the comparison results on communication cost of these schemes are listed in Table 4.

For the communication costs of *l* attributes, Wang et al.’s scheme [20] includes (4l+3) the element’s length in G and one the element’s length in $G_{T}$. Therefore, the total communication cost is 256l+576 bytes. Emura et al.’s scheme [21] includes (3l+2) the element’s length in G and one the element’s length in $G_{T}$. Therefore, the total communication cost is 192l+512 bytes. Hu et al.’s scheme [22] includes (2l+3) the element’s length in G and one the element’s length in $G_{T}$. Therefore, the total communication cost is 128l+576 bytes. Rao et al.’s scheme [23] includes (2l+4) the element’s length in G and one the element’s length in $G_{T}$. Therefore, the total communication cost is 128l+640 bytes. The proposed scheme includes (l+4) the element’s length in G, one the element’s length in $G_{T}$ and the outputs length of one-way hash function in cuckoo filter. Therefore, the total communication cost is 84l+640 bytes.

**Figure 6:**
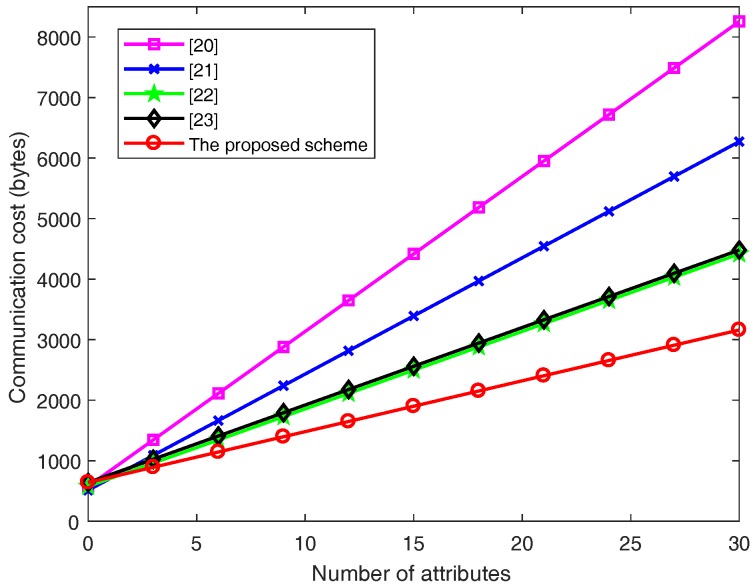
Unsigncrypt cost with the number of attributes.

Figure 6 demonstrates the relationship between the communication cost and the number of attributes.

From Figure 6, the growth of the ciphertext size is linear when the number of attributes increases in all schemes. We could intuitively find out that the communication cost of our proposed scheme is much less than that for other schemes. On the other hand, as Figure 6 shows, when the amount of attributes reaches 30, the communication cost of Wang et al.’s scheme [20], Emura et al.’s scheme [21], Hu et al.’s scheme [22] and Rao et al.’s scheme [23] and the proposed scheme is 7956, 6272, 4416, 4480 and 3100 bytes, respectively. Then the proposed scheme is compared with these schemes [20,21,22,23], which can save 61.7%, 57.6%, 28.5%, 29.5% of bandwidth, respectively.

Obviously, although the cuckoo filter is used to hide access policy in this paper, it does not increase communication overhead compared with other schemes. Also, our scheme has the best performance in terms of communication cost in the all five schemes.

In summary, the proposed CP-ABSC scheme achieves low computation and communication cost, which is comparatively more suited to the EHR system.

## 8. Conclusions

The proposed scheme provides the secure access control of the EHR data as well as prevents the personal privacy information of EHR owners will not be leaked from the LSSS access policy. We show that the proposed scheme is provably security in the standard model under the *q*-DBDHE assumption and *q*-CDHE assumption. Detailed performance analysis results indicate that the proposed scheme has lower computation costs and communication overheads than the related schemes. In addition, the proposed scheme protects the EHR owners’ sensitive privacy information and is more suitable for EHR system. In the future, we would like to focus on how to design another scheme, such as security and efficient of PPAC scheme without bilinear pairing in EHR system.

## Figures and Tables

**Table 1:** Comparison of computation cost.

Scheme	KP/CP	Access Structure	Public Verifiability	MC	CU	Security Model	Privacy-Preserving
[20]	CP	Monotone tree	No	CPA	CMA	ROM	No
[21]	CP	Monotone tree	Yes	CCA	CMA	SM	No
[22]	KP	Threshold policy	No	CCA	CMA	SM	No
[23]	CP	LSSS	Yes	CCA	CMA	SM	No
our	CP	LSSS	Yes	CCA	CMA	SM	Yes

**Table 2:** Time cost of cryptographic operation.

Cryptographic Operation	Execution Time
Bilinear pairing $T_{p}$	9.0791
Scalar multiplication in G $T_{m}$	3.7770
Scalar multiplication in $G_{T}$ $T_{mt}$	0.9243

**Table 3:** Comparison of computation cost.

Scheme	Signcrypt	Unsigncrypt
[20]	7.554l+23.5863 ms	38.165l+37.2407 ms
[21]	15.108l+22.2587 ms	54.4746l+27.2373 ms
[22]	22.662l+23.5683 ms	47.2441l ms
[23]	15.108l+8.4783 ms	20.4101l+52.9495 ms
The proposed scheme	18.885l+27.3633 ms	19.0825l+51.4244 ms

**Table 4:** Comparison of communication costs.

Scheme	*l* Attributes
[20]	256l+576 bytes
[21]	192l+512 bytes
[22]	128l+576 bytes
[23]	128l+640 bytes
The proposed scheme	84l+640 bytes
